# Nitrogen-Bridged Fused-Ring Nonacyclic and Heptacyclic A–D–A Acceptors for Organic Photovoltaics

**DOI:** 10.1021/acsami.4c11466

**Published:** 2024-10-14

**Authors:** Yung-Jing Xue, Yu-Chieh Wang, Han-Cheng Lu, Chia-Lin Tsai, Chia-Fang Lu, Li-Lun Yeh, Yen-Ju Cheng

**Affiliations:** † Department of Applied Chemistry, National Yang Ming Chiao Tung University, 1001 University Road, Hsinchu, Taiwan 30010; ‡ Center for Emergent Functional Matter Science, National Yang Ming Chiao Tung University, 1001 University Road, Hsinchu, Taiwan 30010

**Keywords:** organic photovoltaics, A−D−A type, non-fullerene acceptors, nitrogen-bridged atom, ladder-type structure

## Abstract

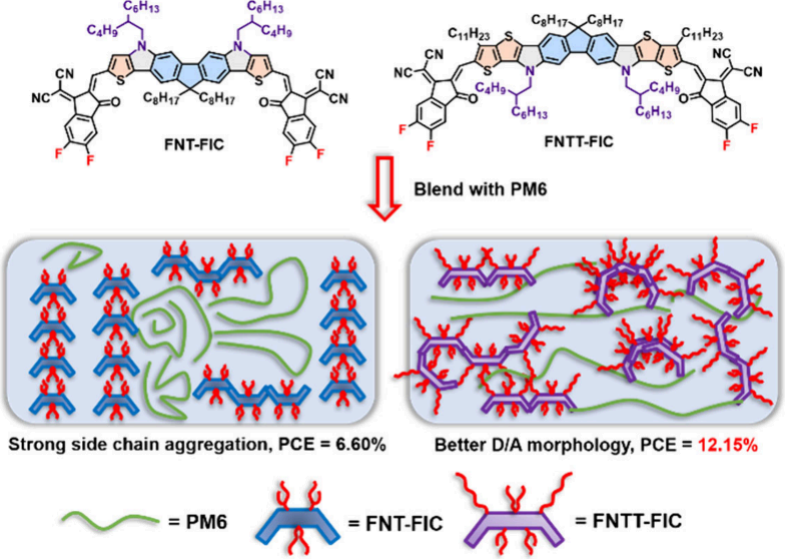

In this work, we designed two nitrogen-bridged fluorene-based heptacyclic FNT and nonacyclic FNTT ladder-type structures, which were constructed by one-pot palladium-catalyzed Buchwald–Hartwig amination. FNT and FNTT were further end-capped by FIC acceptors to form two FNT-FIC and FNTT-FIC non-fullerene acceptors (NFAs), respectively. The two NFAs exhibit more red-shifted absorption and higher crystallinity compared to those of the corresponding carbon-bridged FCT-FIC and FCTT-FIC counterparts. Grazing incidence wide-angle X-ray scattering (GIWAXS) measurements reveal that the 2-butyloctyl groups on the nitrogen in the convex region of FNT-FIC interdigitate with the dioctyl groups on the fluorene in the concave region of another FNT-FIC, resulting in a lamellar packing structure with a *d* spacing of 13.27 Å. As a consequence, the PM6:FNT-FIC (1:1 wt %) device achieved a power conversion efficiency (PCE) of only 6.60%, primarily due to the highly crystalline nature of FNT-FIC, which induced significant phase separation between PM6 and FNT-FIC in the blended film. However, FNTT-FIC, featuring 2-butyloctyl groups positioned on the nitrogen within the concave region of its curved skeleton, exhibits improved donor–acceptor miscibility, thereby promoting a more favorable morphology. As a result, the PM6:FNTT-FIC (1:1.2 wt %) device exhibited a higher PCE of 12.15% with an exceptional *V*_oc_ of 0.96 V. This research demonstrates that placing alkylamino moieties within the concave region of curved A–D–A NFAs leads to a better molecular design.

## Introduction

Over the past few years, organic non-fullerene acceptors (NFAs) have become the primary focus of research in the field of organic photovoltaics (OPVs) owing to their strong absorption covering the visible and near-infrared regions, tunable highest occupied molecular orbital (HOMO) and lowest unoccupied molecular orbital (LUMO) energy levels, and good thermal stability.^1−4^ The commonly designed strategy of organic NFAs is based on an A–D–A (acceptor–donor–acceptor) architecture consisting of a central electron-rich fused-ring donor (D) end-capped by two electron-deficient acceptors (A).^5−14^ Photoinduced intramolecular charge transfer can occur between the push–pull D–A system, imparting the light-harvesting ability on the A–D–A-type NFAs. Molecular rigidification and planarization of the central D cores can elongate effective conjugation, improve charge mobility, and promote intermolecular interactions, which are essential for achieving high-performance NFAs.^15,16^ The formation of a fused-ring fully planarized ladder-type structure typically involves incorporation of sp^3^ carbon bridges to covalently ring-lock adjacent electron-rich aryl groups.^17−25^ Two aliphatic side chains attached at a tetrahedral sp^3^ carbon stick out of the conjugated plane of the ladder-type donor, thus preventing severe aggregation for sufficient solution processability. However, the steric hindrance also inevitably limits the intermolecular interactions and reduces crystallinity.^26,27^

In 2019, Y6-series NFAs have emerged as superior materials due to their enhanced absorption ability and crystallinity.^28−31^ The most distinct feature of Y6 derivatives compared to typical A–D–A structures is the use of sp^2^–nitrogen bridges, which provides several advantages that eventually lead to highly efficient OPV performance.^32−47^ (1) The lone pair electrons on the nitrogen can delocalize along the π-electron system, extending the conjugated length. (2) The stronger electron-donating ability of nitrogen can further enhance intramolecular charge transfer (ICT) for red-shifted absorption. (3) Having only one aliphatic side chain on a bridge atom reduces steric hindrance, facilitating π–π stacking and improved crystallinity. Cadogan cyclization is commonly used to synthesize nitrogen-bridged fused-ring structures. However, the synthesis of the precursor nitro compounds required for intramolecular Cadogan cyclization is challenging, and many functional groups cannot withstand the harsh reaction conditions during cyclization.^48^ As a result, the synthesis of nitrogen-bridged ladder-type cores remains difficult.

**Figure 1:**
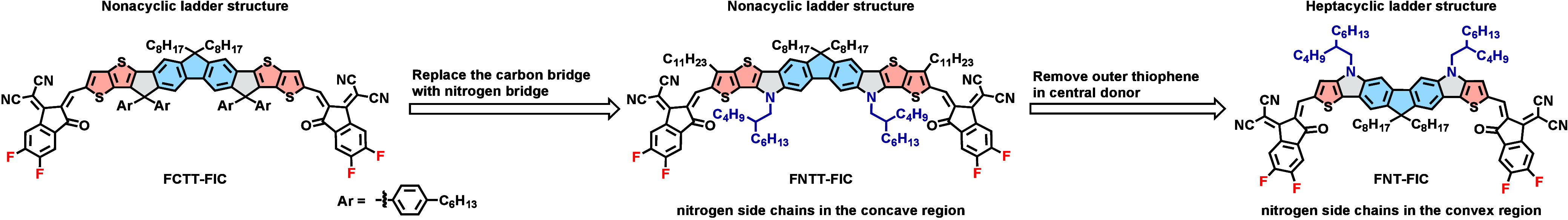
Molecular design and chemical structures of FNT-FIC and FNTT-FIC.

We previously developed an A–D–A-type NFA denoted as FCTT-FIC consisting of a nonacyclic ladder-type D core (FCTT) where the central fluorene unit is fused with two thieno[3,2-*b*]thiophene (TT) units by two sp^3^ carbon bridges (Figure 1).^49^ A nitrogen-bridged version of FCTT-FIC would be highly desirable, as would a systematic investigation of the bridge atom effect. To this end, we further designed and synthesized a nonacyclic nitrogen-bridged FNTT in which two sp^2^–nitrogen bridges are used to fuse the central fluorene unit with two outer β-alkylated TT units. In addition, upon replacement of the TT units with thiophene (T) units, a heptacyclic FNT was also developed for comparison. FNTT and FNT, in which the two pyrrole units were constructed by one-pot palladium-catalyzed Buchwald–Hartwig amination, were further condensed with FIC acceptors to form A–D–A-type FNTT-FIC and FNT-FIC, respectively (Figure 1).

By virtue of noncovalent interactions between the carbonyl groups and the terminal thiophene units to intramolecularly lock the conformation of the FIC acceptor, the FNTT-FIC and FNT-FIC backbone adopts a curved C-shaped geometry with *C*_2*v*_ symmetry.^50^ The side chains attached to the pyrrole units in FNT-FIC are located in the convex region, whereas the nitrogen side chains in FNTT-FIC are situated in the concave region. The side chain location in C-shaped A–D–A-type NFAs, whether in the convex or concave regions, may play a key role in molecular assembly. Nitrogen-bridged FNTT-FIC and FNT-FIC show more red-shifted absorption spectra and enhanced crystallinity compared with those of their carbon-bridged counterparts. FNT-FIC self-assembles into a layer structure composed of consecutive C-shaped molecules connected through side chain interdigitation. The 2-butyloctyl groups on the nitrogen in the convex region of FNT-FIC interact with the dioctyl groups on fluorene in the concave region of another FNT-FIC, forming a lamellar packing structure. The PM6:FNT-FIC (1:1 wt %) device delivered a moderate power conversion efficiency (PCE) of only 6.60% due to the highly crystalline nature of FNT:FIC that caused severe phase separation between PM6 and FNT-FIC in the blended film. However, FNTT-FIC containing six side chains on the FNTT ladder core is more soluble and, thus, shows a more favorable D–A mixed morphology. As a result, the PM6:FNTT-FIC (1:1.2 wt %) device exhibited a higher PCE of 12.15%.

## Experimental Section

### Materials

All reagents and chemicals were purchased from commercial sources and used without further purification unless noted otherwise. PM6 was purchased from Solarmer, Inc. The synthetic details of FNT-FIC and FNTT-FIC and fabrication of OPV devices can be found in the Supporting Information.

## Results and Discussion

### Synthesis and Characterization of Materials

**Scheme 1:**
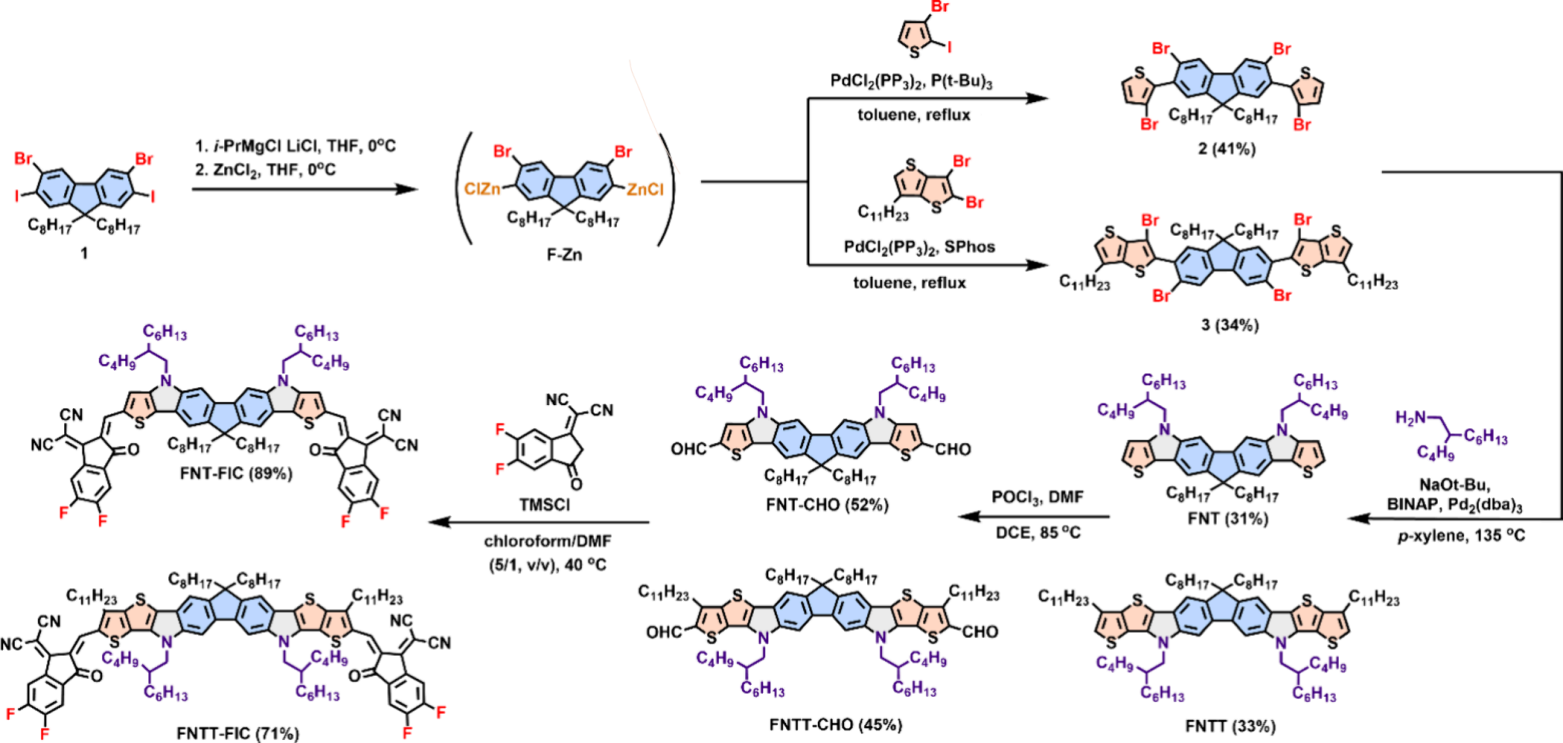
Synthetic Routes for FNT-FIC and FNTT-FIC

The synthetic routes of the two new materials, FNT-FIC and FNTT-FIC, are shown in Scheme 1. Compound **1** was synthesized according to our previous work.^51^ Grignard metathesis of **1** with isopropyl magnesium chloride occurred regioselectively at positions 2 and 7 of fluorene due to the higher reactivity of iodine atoms followed by quenching with ZnCl_2_ to form the F–Zn intermediate. Palladium-catalyzed Negishi coupling of 2-iodo-3-bromothiophene or 2,3-dibromo-6-undecylthieno[3,2-*b*]thiophene with the freshly prepared F–Zn formed compounds **2** and **3**, respectively. Palladium-catalyzed Buchwald–Hartwig amination of **2** and **3** with 2-butyloctylamine resulted in the formation of two pyrrole rings in FNT and FNTT, respectively. FNT and FNTT were formylated with DMF/POCl_3_ Vilsmeier reagent to form FNT-CHO and FNTT-CHO which were further condensed with FIC to complete the synthesis of FNT-FIC and FNTT-FIC, respectively. The detailed synthetic procedure, mass spectrometry, and ^1^H nuclear magnetic resonance (NMR) and ^13^C NMR spectra of the new compounds are provided in the Supporting Information (Figure S8–S23).

### Optical and Electrochemical Properties

**Figure 2:**
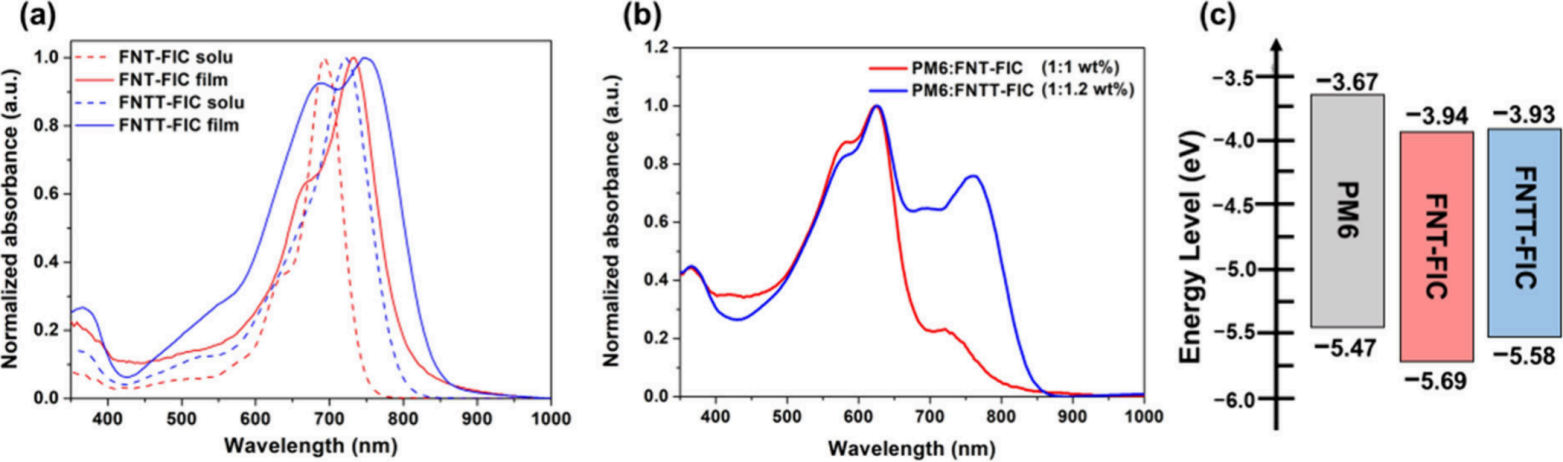
(a) Absorption spectra of FNT-FIC and FNTT-FIC in chloroform, thin films, and (b) PM6:FNT-FIC (1:1 wt %) and PM6:FNTT-FIC (1:1.2 wt %) blended films. (c) HOMO and LUMO energy level diagram of PM6, FNT-FIC, and FNTT-FIC estimated by cyclic voltammetry.

The normalized absorption spectra of two materials are shown in Figure 2, and their intrinsic properties are listed in Table 1. In the solution state, FNT-FIC and FNTT-FIC exhibit absorption maxima (λ_max_) at 692 and 722 nm with extinction coefficients of 1.89 × 10^5^ and 2.06 × 10^5^, respectively (Figure S1). The more red-shifted λ_max_ of FNTT-FIC is attributed to the greater conjugation length of the central nonacyclic π-core incorporating thieno[3,2*-b*]thiophene (TT) as the outermost units. From the solution to the thin film state, the absorption λ_max_ values of FNT-FIC and FNTT-FIC are red-shifted to 733 nm (Δλ_max_ = 51 nm) and 746 nm (Δλ_max_ = 24 nm), respectively, indicative of stronger intermolecular π–π interactions in FNT-FIC. Notably, FNTT-FIC exhibits another blue-shifted absorption peak centered at 689 nm. The presence of bathochromic and hypochromic bands relative to the original peak in the solution state suggests the coexistence of *J* aggregation and *H* aggregation in the thin film state.^52−54^ In the blended film absorption spectra (Figure 2b), the PM6:FNTT-FIC film displays significantly broader and stronger absorption in the range of 680–850 nm, enabling an increased level of light harvesting and, thereby, generating more photocurrent.

**Table 1:** Summary of the Intrinsic Properties of FNT-FIC and FNTT-FIC

		λ_max_ (nm)							
NFA	extinction coefficient (×10^5^ cm^–1^ M^–1^)^a^	solution	film	Δλ (nm)	λ_onset_ (nm)^b^	*E*_opt,g_ (eV)^c^	HOMO (eV)^d^	LUMO (eV)^d^	*E*_ele,g_ (eV)^d^
FNT-FIC	1.89	692	733	51	796	1.56	–5.69	–3.94	1.75
FNTT-FIC	2.06	722	746, 689	24	834	1.49	–5.58	–3.93	1.65

a Calculated at λ_max_ in the solution state.

b Calculated in the solid state.

c *E*_opt,g_ = 1240/λ_onset_.

d Determined by cyclic voltammetry.

The HOMO and LUMO energy levels of two materials were determined by cyclic voltammetry (Figure S2 and eqs S1 and S2). FNT-FIC and FNTT-FIC show similar LUMO energy levels of −3.94 and–3.93 eV, respectively, whereas the HOMO energy level of FNT-FIC (−5.69 eV) is lower than that of FNTT-FIC (−5.58 eV). The higher HOMO energy level of FNTT-FIC indicates that embedding the TT unit in the nonacyclic ladder core strengthens the electron-donating capability.

### Thermal Properties

**Figure 3:**
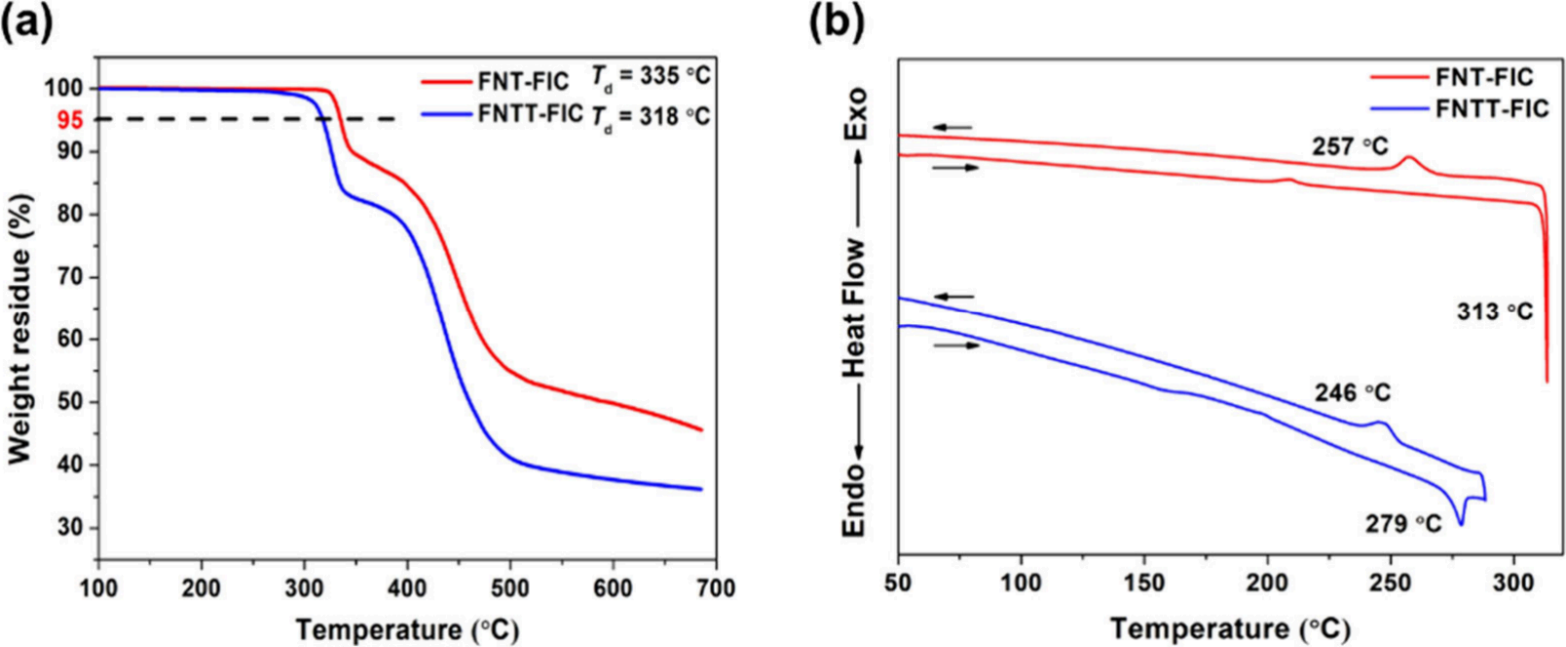
(a) TGA and (b) DSC measurements of FNT-FIC and FNTT-FIC with a ramping rate of 10 °C/min.

Thermal properties were measured by thermogravimetric analysis (TGA) and differential scanning calorimetry (DSC). In Figure 3, FNT-FIC and FNTT-FIC show decomposition temperatures (*T*_d_) of 335 and 318 °C, respectively, indicating good thermal stability. In the DSC measurement, the melting points (*T*_m_) for FNT-FIC and FNTT-FIC upon heating are 313 and 279 °C, respectively, while the crystallization temperatures (*T*_c_) upon cooling are 257 and 246 °C, respectively. The higher *T*_m_ and *T*_c_ values of FNT-FIC suggest stronger intermolecular interactions, which are consistent with the absorption results.

### Theoretical Calculations

**Figure 4:**
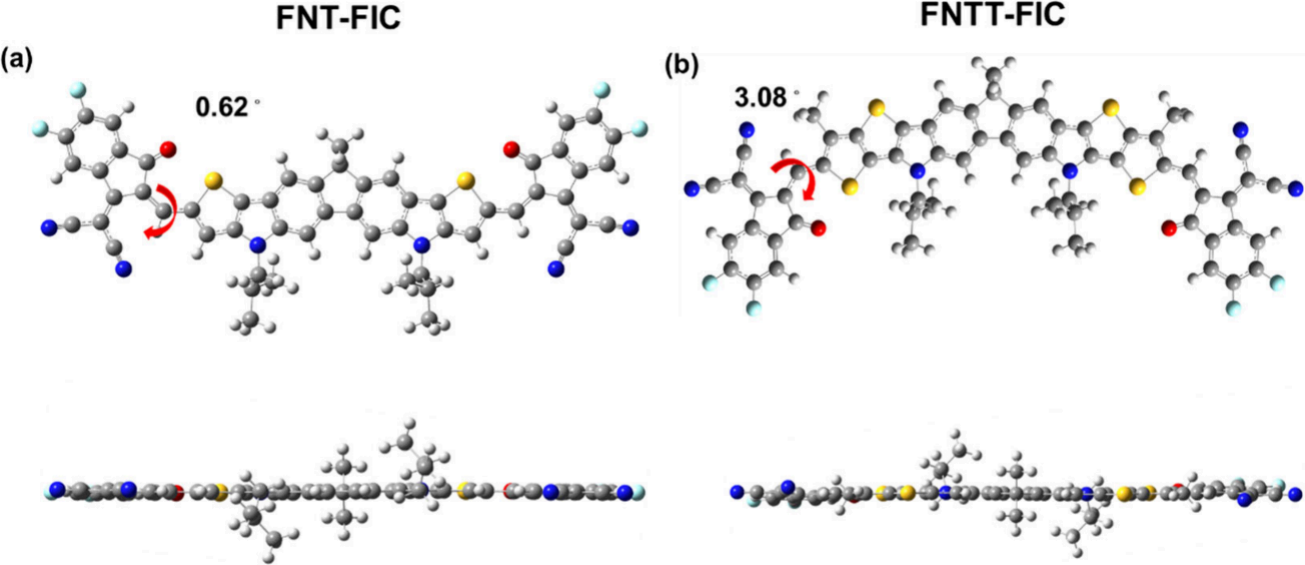
Top and side views of the optimized conformation of (a) FNT-FIC and (b) FNTT-FIC calculated at the B3LYP/6-311G(d,p) level.

Density functional theory (DFT) calculation with Gaussian09 suite15 at the B3LYP/6-311G(d,p) level was employed to investigate the molecular geometry and dipole moment of the NFAs. FNT-FIC and FNTT-FIC with simplified side chains (the undecyl and 2-butyloctyl groups are simplified to methyl and 2-methylpropyl groups, respectively) are used for simulation. The optimal geometries of the FNT-FIC and FNTT-FIC structures are shown in Figure 4. The dihedral angles between the π-core and end group of FNT-FIC and FNTT-FIC are 0.62° and 3.08°, respectively. Due to the steric hindrance of the side chain on the TT unit, the structure of FNTT-FIC is more twisted than that of FNT-FIC, which may weaken intermolecular interactions.

The HOMO and LUMO energy levels of the two materials are plotted in Figure S3. The electrons are delocalized throughout central ladder core in the HOMO, while the electrons are further delocalized to terminal group acceptors in the LUMO. The HOMO and LUMO energy levels (−5.66 and–3.65 eV, respectively) of FNTT-FIC are higher than those of FNT-FIC (−5.86 and–3.71 eV, respectively). The trend in the HOMO and LUMO energy levels of FNT-FIC and FNTT-FIC obtained from theoretical calculations aligns well with the experimental results.

### Device Performance

**Figure 5:**
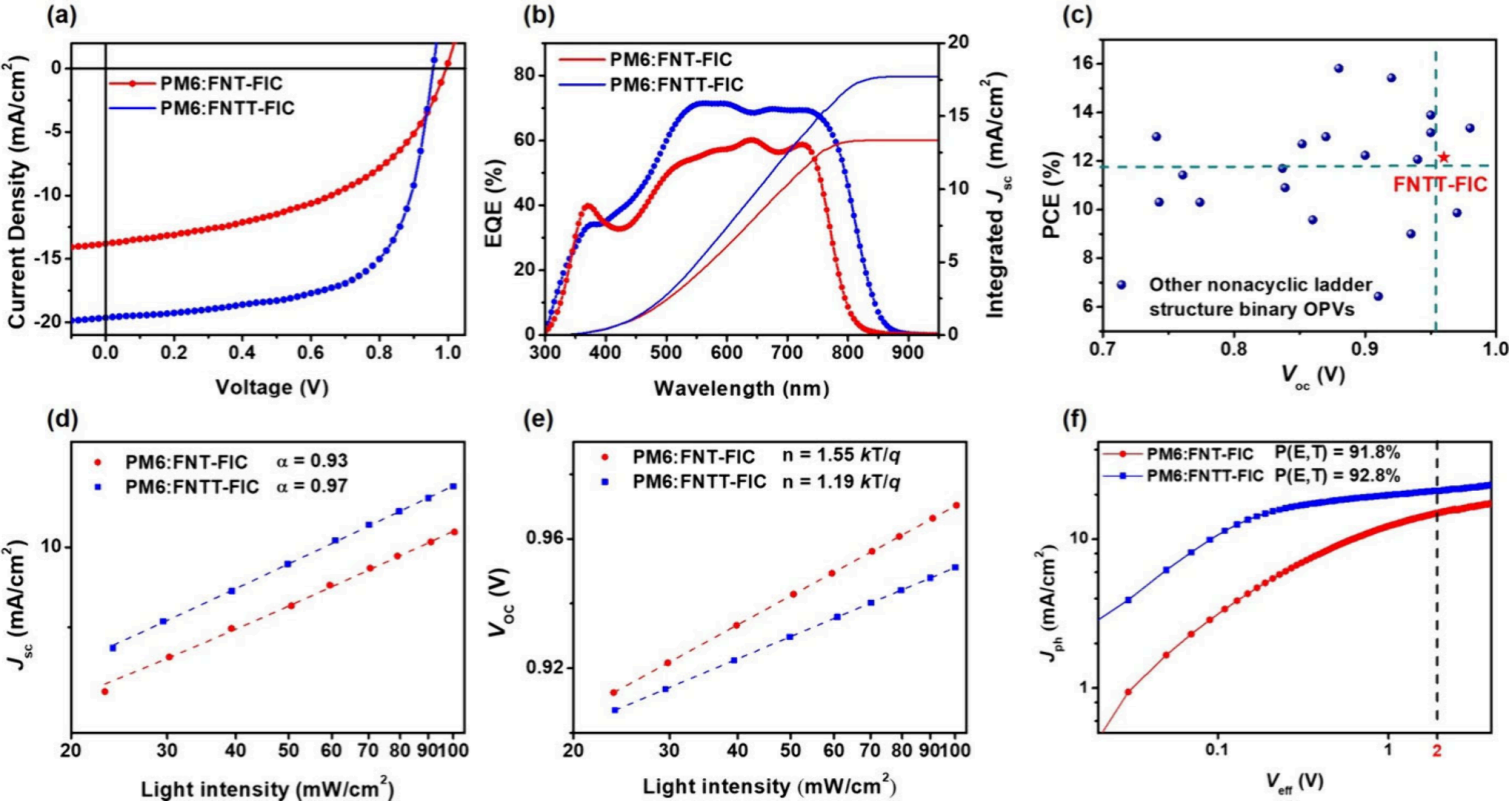
(a) *J*–*V* curves and (b) EQE spectra of the PM6:FNT-FIC and PM6:FNTT-FIC devices. (c) Plots of *V*_oc_ vs PCE in binary OPVs using nonacyclic NFAs reported in the literature (11 papers) in comparison with those of FNTT-FIC in this work. (d) *J*_sc_ vs light intensity and (e) *V*_oc_ vs light intensity of the FNT-FIC- and FNTT-FIC-based devices. (f) *J*_ph_ vs *V*_eff_ of the optimized devices.

The inverted (ITO/ZnO/PM6:NFA/MoO_3_/Ag) OPV devices were fabricated with the PM6 polymer as the p-type material. The characteristics of these devices, including the *J*–*V* curve and the external quantum efficiency (EQE) spectra, are shown in Table 2 and panels a and b of Figure 5. The optimized PM6:FNT-FIC (1:1 wt %) device, using chlorobenzene as the solvent and thermal annealing (TA) at 120 °C, exhibited a low PCE of 6.60%, with a *V*_oc_ of 0.99 V, a *J*_sc_ of 13.81 mA/cm^2^, and a fill factor (FF) of 48.21%. In contrast, the optimized PM6:FNTT-FIC (1:1.2 wt %) device, using the nonhalogenated solvent *o*-xylene without TA, showed a higher PCE of 12.15%, with a *V*_oc_ of 0.96 V, a *J*_sc_ of 19.64 mA/cm^2^, and an FF of 64.52%. It is noteworthy that the *V*_oc_ value is among the highest reported for nitrogen-based nonacyclic structure NFAs used in binary OPVs in the literature (Figure 5c and Table S1). The higher *J*_sc_ of the FNTT-FIC-based device is ascribed to the significantly broader absorption range of the FNTT-FIC, as demonstrated by the EQE spectrum.

**Table 2:** Characteristics of the ITO/ZnO/Active Layer/MnO_3_/Ag Devices

active layer	blend ratio (wt %)	*V*_oc_ (V)	*J*_sc_ (mA/cm^2^)	FF (%)	PCE (%)^c^
PM6:FNT-FIC^a^	1:1	0.99 (0.99 ± 0)	13.81 (13.65 ± 0.17)	48.21 (46.52 ± 0.74)	6.60 (6.28 ± 0.15)
PM6:FNTT-FIC^b^	1:1.2	0.96 (0.96 ± 0)	19.64 (19.41 ± 0.21)	64.52 (63.13 ± 0.73)	12.15 (11.74 ± 0.21)

a Fabricated using 1.5 wt % DIO in chlorobenzene as the processing solvent and annealing at 120 °C for 5 min.

b Fabricated using 0.5 wt % DIO in *o*-xylene as the processing additive without annealing.

c The average values with the standard deviation over 10 cells are shown in parentheses.

### Charge Recombination Analysis

To deeply investigate the charge recombination, we examined the *J*_sc_ versus light intensity, and the results are shown in Figure 5d. The α value in *J*_sc_ ∝ *P*_light_^α^, where *P*_light_ is the light intensity, is indicative of bimolecular recombination. The α value is close to 1, indicating that most free carriers are collected at the electrodes prior to recombination.^55,56^ The α values for the PM6:FNT-FIC and PM6:FNTT-FIC devices are 0.93 and 0.97, respectively. These results illustrate that bimolecular recombination is suppressed in the PM6:FNTT-FIC device. Plots of *V*_oc_ versus light intensity are shown in Figure 5e. The *n* values, obtained from the equation *V*_oc_ = *nkT* ln(*P*_light_)/*q*, where *k* is the Boltzmann constant, *T* is the absolute temperature, and *q* is the electron charge, were determined to be 1.55 and 1.19 *kT*/*q* for the PM6:FNT-FIC and PM6:FNTT-FIC devices, respectively. The *n* value being close to 1 suggests that the PM6:FNTT-FIC device effectively reduces the trap-assisted recombination pathway in the binary system.^57^

To understand the exciton dissociation of the two devices, we plotted the photocurrent density (*J*_ph_) versus the effective voltage (*V*_eff_) of the device, where *J*_ph_ can be obtained by subtracting the dark current density from the illuminated current density and *V*_eff_ is the voltage at *J*_ph_ = 0 minus the applied bias voltage.^58,59^ The relationships between *J*_ph_ and *V*_eff_ are shown in Figure 5f. When *V*_eff_ is >2 V, *J*_ph_ reaches the saturation current (*J*_sat_), at which charge recombination is minimized because of the high internal electric field. Charge dissociation probability *P*(*E*, *T*) of devices, which can be obtained from the *J*_sc_/*J*_sat_ ratio under short current conditions, are 91.8% and 92.8% for PM6:FNT-FIC and PM6:FNTT-FIC, respectively. The higher *P*(*E*, *T*) indicates better charge dissociation, which is beneficial for enhanced *J*_sc_ and FF values in the device.

### Space-Charge-Limited Current Measurements

The electron and hole mobilities of PM6:FNT-FIC (μ_h_ = 9.81 × 10^–7^ cm^2^ V^–1^ s^–1^, and μ_e_ = 2.27 × 10^–7^ cm^2^ V^–1^ s^–1^) and PM6:FNTT-FIC (μ_h_ = 8.42 × 10^–5^ cm^2^ V^–1^ s^–1^, and μ_e_ = 1.06 × 10^–4^ cm^2^ V^–1^ s^–1^) were evaluated by space-charge-limited current (SCLC) methods (Figure S4 and eq S3).^60^ Indeed, PM6:FNTT-FIC exhibited higher μ_h_ and μ_e_ values with a more balanced μ_e_/μ_h_ ratio of 1.26, which is consistent with the observed higher *J*_sc_ and FF values. The improved electron and hole charge mobilities suggest a uniform donor–acceptor network structure of the PM6:FNTT-FIC blend for better carrier transport. In contrast, the low μ_h_ and μ_e_ values of PM6:FNT-FIC were attributed to severe phase segregation caused by the high crystallinity of FNT-FIC.

### GIWAXS Analysis

**Figure 6:**
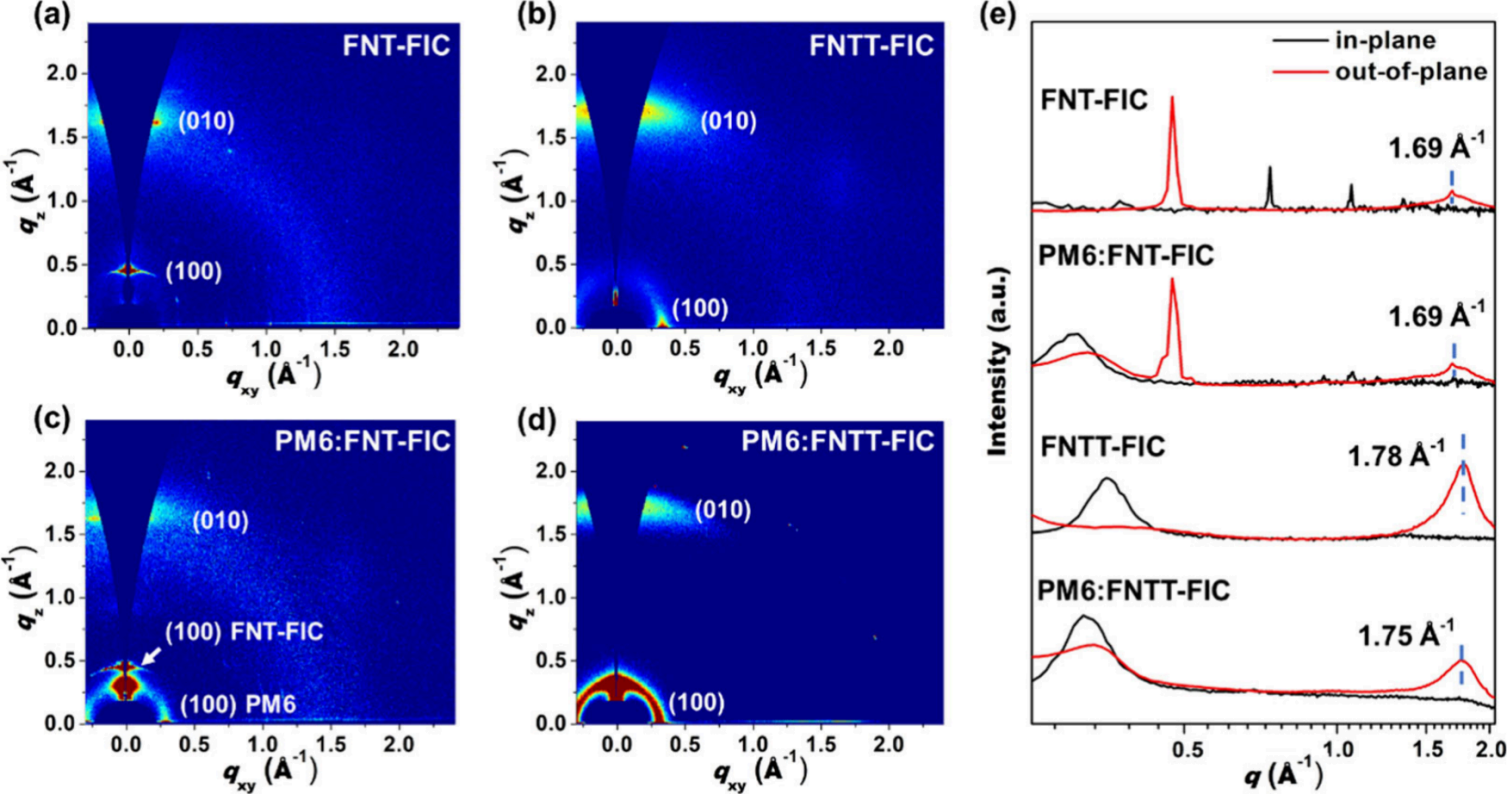
Two-dimensional GIWAXS of (a) FNT-FIC and (b) FNTT-FIC neat films and (c) PM6:FNT-FIC and (d) PM6:FNTT-FIC blended films. (e) Corresponding one-dimensional line-cut profiles along the in-plane and out-of-plane directions.

**Figure 7:**
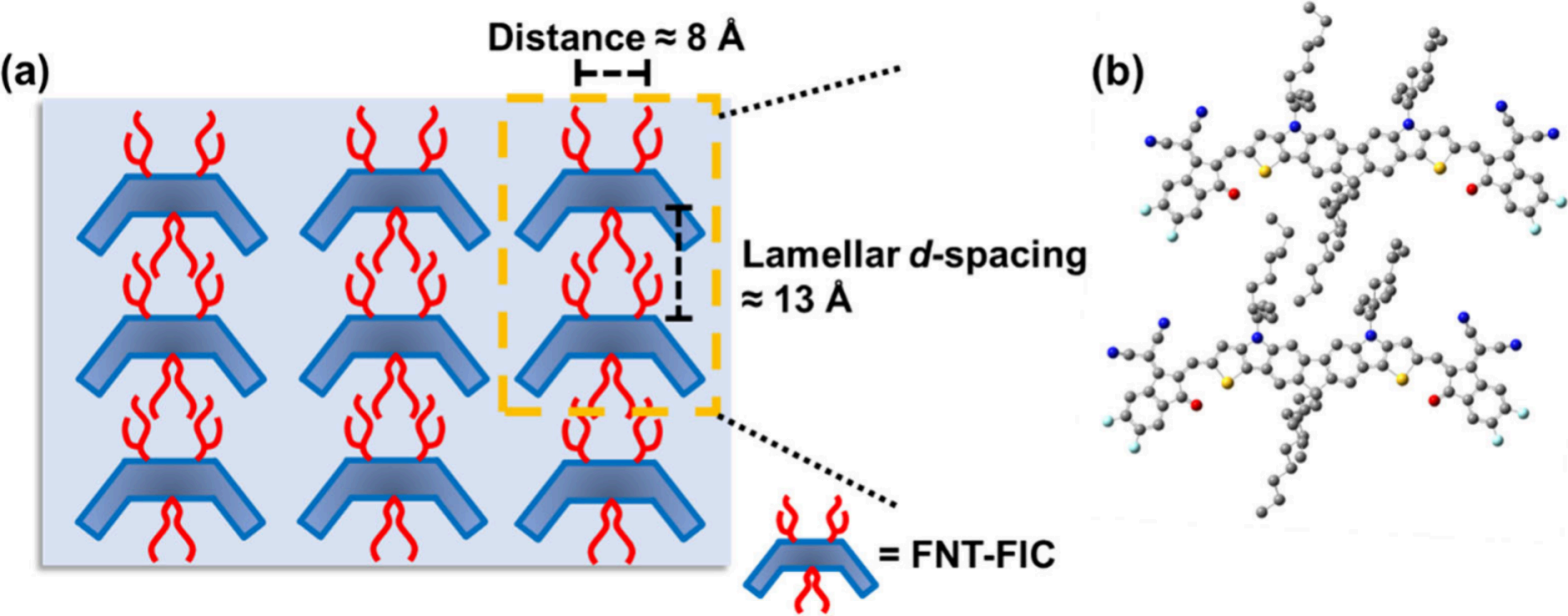
(a) Schematic illustration of the proposed lamellar molecular packing of FNT-FIC with a *d* spacing of ∼13 Å along the out-of-plane direction. (b) Simulation of two molecules packing via side chain interdigitation.

Grazing incidence wide-angle X-ray scattering (GIWAXS) was used to study the molecular packing of neat NFAs and the films blended with PM6. The two-dimensional diffraction images and corresponding one-dimensional in-plane (*q*_*xy*_) and out-of-plane (*q*_*z*_) patterns (Figure S5) of the thin films are illustrated in Figure 6, and the structural parameters are listed in Table 3. The FNT-FIC (Figure 6a) neat file displays π–π stacking diffraction at a *q*_*z*_ of 1.69 Å^–1^ with a *d* spacing (*d*_*π*_) of 3.72 Å, indicating a typical face-on stacking orientation. Additionally, we observed a sharp lamellar diffraction peak at a *q*_*z*_ of 0.47 Å^–1^ corresponding to an edge-on orientation with a short *d* spacing (*d*_l_) of 13.27 Å, which is rarely seen in typical NFAs. These results indicate the coexistence of both face-on and edge-on orientations in the FNT-FIC neat film. Judging from the structure of FNT-FIC, we envisage that the two 2-butyloctyl groups on the nitrogen bridges in one molecule can penetrate the bay area of another molecule. The distance between two 2-butyloctyl groups is ∼8 Å, allowing them to sandwich the 9,9-dioctyl groups on the fluorene unit in the other FNT-FIC molecule. This arrangement could lead to a layered structure consisting of a consecutive C-shaped molecular assembly through side chain interdigitation along the out-of-plane direction. Theoretical calculations of such a molecular assembly, as illustrated in Figure 7, confirm that the lamellar spacing is ∼13 Å. which is consistent with the GIWAXS result. The location of the nitrogen side chains in the convex region of FNT-FIC enabled the consecutive self-assembly of the aliphatic chains. On the basis of the Scherrer equation, the coherence length (*L*_c_) of the sharp lamellar diffraction indicates a crystalline domain of 29.7 nm, which is significantly larger than the average exciton diffusion length of organic semiconductors.^61^ In addition, two sharp peaks at *q*_*xy*_ values of 0.74 and 1.07 Å^–1^ were observed, which can be ascribed to periodic lamellar spacings of 8.49 and 5.87 Å, respectively, between adjacent curved FNT-FIC molecules along the in-plane direction (Figure 7).

**Table 3:** Detailed GIWAXS Data of Neat and Blended Films

	in-plane diffraction peak				out-of-plane diffraction peak			
material	*q*_*xy*_^a^ (Å^–1^)	*d*^b^ (Å)	fwhm^c^ (Å^–1^)	*L*_c_^d^ (Å)	*q*_*z*_^a^ (Å^–1^)	*d*^b^ (Å)	fwhm^c^ (Å^–1^)	*L*_c_^d^ (Å)
FNT-FIC	0.74	8.49	0.01	377.96	0.47	13.27	0.02	296.53
	1.07	5.87	0.01	377.96	1.69	3.72	0.25	22.56
FNTT-FIC	0.35	17.88	0.08	70.52	1.78	3.54	0.22	25.14
PM6:FNT-FIC	0.31	20.32	0.09	65.17	0.47	13.27	0.02	296.53
					1.69	3.72	0.28	20.05
PM6:FNTT-FIC	0.32	19.87	0.09	65.39	1.75	3.58	0.35	16.19

a Obtained from original data.

b Calculated from the equation *d*_π_ = 2π/*q*.

c Obtained from the fitted patterns of line-cut profiles.

d Calculated from the Scherrer equation *L*_C_ = 2π*k*/fwhm, where fwhm is the full-width at half-maximum of the peak and *k* is a shape factor (we use 0.9).

The lamellar diffraction at a *q*_*z*_ of 0.47 Å^–1^ (*d*_l_ = 13.27 Å) and π–π stacking diffraction at a *q*_*z*_ of 1.69 Å^–1^ (*d*_π_ = 3.72 Å) are still found in the PM6:FNT-FIC blended film (Figure 6c). Additionally, an in-plane diffraction at a *q*_*xy*_ of 0.31 Å^–1^ with a *d*_l_ of 20.32 Å attributed to the lamellar stacking of PM6 is observed because a similar peak at a *q*_*xy*_ of 0.29 Å^–1^ is also shown in the PM6 neat film in Figure S6. These results indicate severe phase separation between the two components in the blend film, leading to a weaker donor–acceptor interface for charge separation that could be responsible for the lower *J*_sc_ and FF in the PM6:FNT-FIC device.

The FNTT-FIC neat film (Figure 6b) shows π–π stacking diffraction at a *q*_*z*_ of 1.78 Å^–1^ with a *d*_π_ of 3.54 Å and a lamellar diffraction at a *q*_*xy*_ of 0.35 Å^–1^ with a *d*_l_ of 17.88 Å. No lamellar layer structure assembled along the out-of-plane direction is observed. The presence of six side chains on the nonacyclic FNTT core precludes the formation of the highly crystalline lamellar packing structure observed in FNT-FIC. The PM6:FNTT-FIC blend film (Figure 6d) exhibits similarly a π–π stacking diffraction at a *q*_*z*_ of 1.75 Å^–1^ (*d*_π_ = 3.58 Å) and a lamellar stacking diffraction at a *q*_*xy*_ of 0.32 Å^–1^ (*d*_l_ = 19.87 Å). The slightly increased packing distances suggest better association of FNTT-FIC with PM6, leading to a favorable D–A intermixed morphology to facilitate charge transport for better *J*_sc_ and FF values.

### Atomic Force Microscopy Measurements

**Figure 8:**
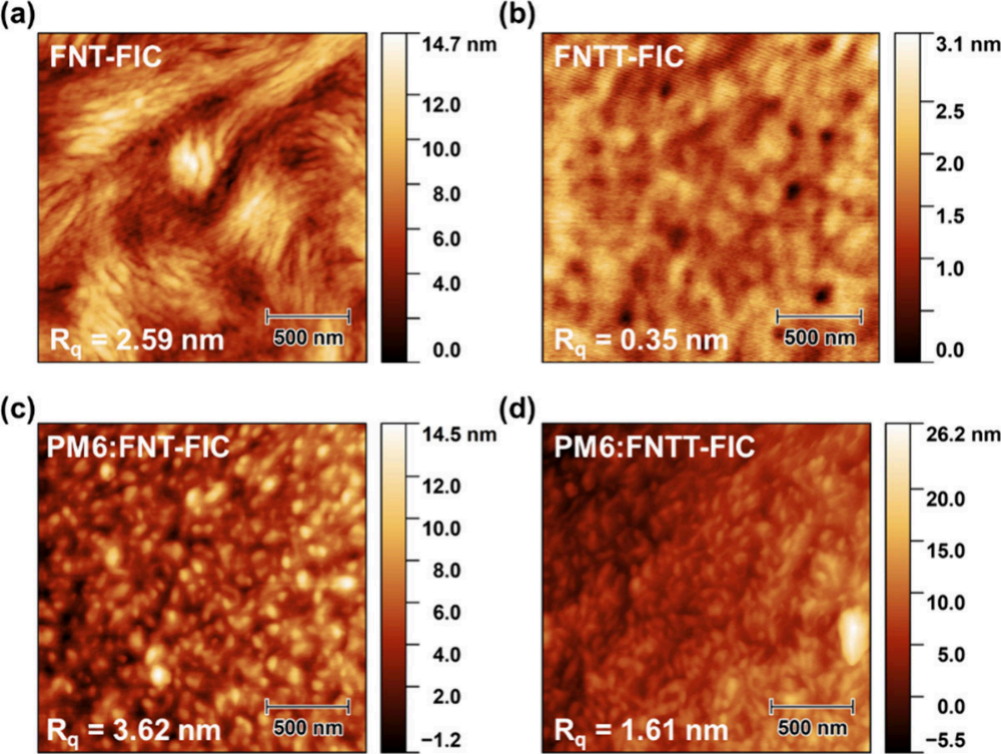
Atomic force microscopy images with roughness of (a) FNT-FIC, (b) FNTT-FIC, (c) PM6:FNT-FIC, and (d) PM6:FNTT-FIC thin films.

Atomic force microscopy (AFM) was used to examine the surface morphologies of the neat and blended films, as depicted in Figure 8. FNT-FIC exhibited substantial aggregation, resulting in a notably high root-mean-square roughness (*R*_q_) of 2.59 nm. In contrast, the FNTT-FIC film displayed a relatively smooth surface with a roughness of 0.35 nm. The *R*_q_ values of the PM6:FNT-FIC and PM6:FNTT-FIC films are 3.62 and 1.61 nm, respectively. The higher *R*_q_ value of PM6:FNT-FIC reveals pronounced phase segregation. In contrast, the lower *R*_q_ value of the PM6:FNTT-FIC film indicates a stronger inclination toward improved complexation and networking with PM6, forming a smoother morphology. These observations align well with the outcomes derived from the GIWAXS analysis.

### Contact-Angle Measurement

To investigate donor–acceptor miscibility, contact-angle measurements were performed. The Flory–Huggins interaction parameter (χ) was calculated from the surface energy values. Figure S7 shows the droplet contact angles of water and ethylene glycol on neat films of PM6 and NFAs. The surface energies of the PM6, FNT-FIC, and FNTT-FIC neat films were 29.6, 41.6, and 38.4 mN/m, respectively. Consequently, the PM6:FNT-FIC and PM6:FNTT-FIC pairs exhibited χ values of 1.01 and 0.57, respectively. The higher χ value for the PM6:FNT-FIC film indicates weak interaction between PM6 and FNT-FIC, facilitating self-aggregation and phase separation. In contrast, the lower χ value for PM6 reveals better donor–acceptor miscibility, which enhances complexing and networking with PM6 in the thin film, consistent with the GIWAXS and AFM results.

## Conclusions

Non-fullerene acceptors (NFAs) composed of sp^2^–nitrogen bridged ladder cores have emerged as a new generation of n-type materials. In this work, we have developed two nitrogen-bridged fluorene-based heptacyclic FNT and nonacyclic FNTT ladder-type structures constructed by one-pot palladium-catalyzed Buchwald–Hartwig amination. FNT and FNTT were further end-capped by FIC acceptors to form FNT-FIC and FNTT-FIC NFAs, respectively. The two NFAs exhibit more red-shifted absorption and higher crystallinity compared to those of the corresponding carbon-bridged FCT-FIC and FCTT-FIC counterparts. FNTT-FIC exhibits a smaller optical bandgap and a higher HOMO energy level compared to those of FNT-FIC. However, FNT-FIC exhibits strong intermolecular interactions and higher crystallinity. On the basis of the GIWAXS analysis, the 2-butyloctyl groups on the nitrogen in the convex region of FNT-FIC self-assemble with the dioctyl groups on the fluorene in the concave region of another FNT-FIC, forming a lamellar layer packing structure with a *d* spacing of 13.27 Å. As a result, the PM6:FNT-FIC (1:1 wt %) device delivered a PCE of only 6.60% due to the strong self-aggregation of FNT-FIC that caused severe phase separation between PM6 and FNT-FIC in the blended film. FNTT-FIC with 2-butyloctyl groups attached to the nitrogen in the concave region of the curved skeleton shows better D–A miscibility and thus a more favorable D–A intermixed morphology. As a result, the PM6:FNTT-FIC (1:1.2 wt %) device processed by nonhalogenated *o*-xylene without thermal annealing exhibited a higher PCE of 12.15%, an exceptional *V*_oc_ of 0.96 V, and an improved *J*_sc_ of 19.64 mA/cm^2^. This work demonstrates that the location of nitrogen side chains on the curved molecular skeleton plays a crucial role in determining the molecular packing. Positioning alkylamino moieties within the concave region of curved A–D–A NFAs is a superior molecular design.

## Supporting Information Available

Instruments and characterization, synthetic procedures, cyclic voltammograms, DFT calculation of frontier molecular orbitals, fabrication and characterization of OPV devices, SCLC characteristics, GIWAXS film measurements, contact angle measurements, and ^1^H and ^13^C NMR spectra (PDF)
