# Supplementary material for: Nitrogen-Bridged Fused-Ring Nonacyclic and Heptacyclic A–D–A Acceptors for Organic Photovoltaics
Source: ACS Appl Mater Interfaces. 2024 Oct 14;16(42):57481–90. doi: 10.1021/acsami.4c11466 (PMC11503517; doi:10.1021/acsami.4c11466)
Supplement: Supplementary file 1 — am4c11466_si_001.pdf [file am4c11466_si_001.pdf]

Supporting Information (SI)

Nitrogen-Bridged Fused-Ring Nonacyclic and Heptacyclic A-D-A Acceptors for Organic Photovoltaics

Yung-Jing Xue[a], Yu-Chieh Wang[a], Han-Cheng Lu[a], Chia-Lin Tsai[a], Chia Fang Lu[a], Li-Lun Yeh[a] and Yen-Ju Cheng*[ab]

[a]Department of Applied Chemistry, National Yang Ming Chiao Tung University, 1001 University Road, Hsinchu, Taiwan 30010.

[b]Center for Emergent Functional Matter Science, National Yang Ming Chiao Tung University, 1001 University Road, Hsinchu, Taiwan 30010.

Email: yjcheng@nycu.edu.tw

CONTENT

1. Instruments and characterization S1
2. Synthetic procedure S2
2. UV-vis spectrophotometer..S7
3. Cyclic voltammetry (CV) characteristics..S8
4. Density functional theory (DFT) calculation of frontier molecular orbitals....S9
5. Fabrication and Characterization of OPV Devices S9
6. Space-charge limited current (SCLC) characteristics.................................S11
7. GIWAXS film measurements S12
8. Contact angle measurements S13
9. References...S13
10. ^{1}H and ^{13}C NMR spectra S17

1. Instruments and characterization

^{1}H and ^{13}C NMR spectra were measured using Varian-400 MHz and JEOL-400 MHz instrument spectrometers and obtained in deuterated chloroform ($CDCl_3$) with TMS as internal reference unless otherwise stated, and chemical shifts (δ) are reported in parts per million. The mass spectra of the samples were recorded on JEOL T200-GC high resolution spectrometer using electron impact (EI) or field desorption (FD) method. Differential scanning calorimetry (DSC) and thermogravimetric analysis (TGA) were conducted on a TA Q200 Instrument and a TA TGA55 Instrument under nitrogen atmosphere at heating/cooling rate of 10 °C/min. Surface topography was investigated

using Veeco diInnova AFM and standard tips (Tapping mode; Length: 240 μm; Resonance Frequency: 70 kHz; Spring Constant: 2 N/m).

2. Synthetic procedure

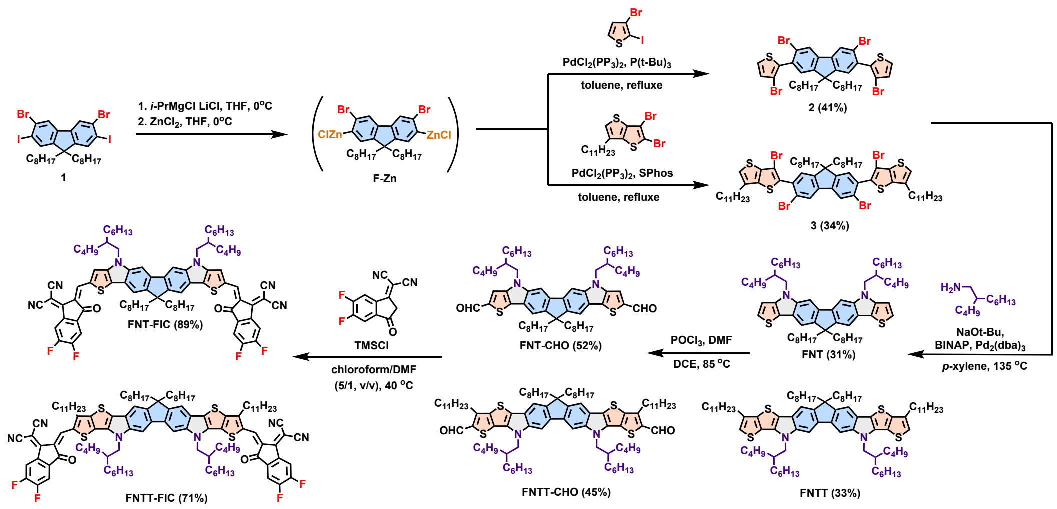

Synthesis of compound 2

To a solution of compound **1** (400 mg, 0.5 mmol) in dry THF (2 mL) under nitrogen was added isopropylmagnesium chloride lithium chloride complex solution (1.3 M in THF, 0.8 mL) dropwise at −78 °C. After stirring at −78 °C for 30 min, the solution of zinc chloride (157 mg, 1.15 mmol) in dry THF (1 mL) was added to the mixture at −78 °C. The reaction mixture was warmed to room temperature and stirred for 1 h to form the F-Zn intermediate. Then, a solution of 2,3-dibromothiophene (433 mg, 1.5 mmol), $Pd(PPh_3)_2Cl_2$ (70 mg, 0.1 mmol) and $P(t\text{-}Bu)_3$ (20 mg, 0.1 mmol) in dry toluene (6 mL) were added with the freshly prepared F-Zn solution slowly at room temperature. The mixture was refluxed for 16 h. After cooling to room temperature, the reaction solution was quenched by NH_4Cl solution and extracted with dichloromethane (25 mL x 2) and water (50 mL). The collection organic layer was dried over $MgSO_4$. After removing the solvent by reduced pressure, the residue was purified by silica gel chromatography with hexane as an eluent to get a light-yellow oil **2** (178 mg, 41%). ^1H NMR (400 MHz,

$CDCl_3$): δ 7.99 (s, 2H), 7.41 (d, J = 5.2 Hz, 2H), 7.37 (s, 2H), 7.10 (d, J = 5.2 Hz, 2H), 1.94 (t, J = 8.0 Hz, 4H), 1.37-0.80 (m, 26H), 0.70 (m, 4H). ^{13}C NMR (100 MHz, $CDCl_3$): δ 150.24, 141.71, 137.50, 132.86, 130.08, 127.44, 126.13, 124.74, 123.44, 111.27, 55.43, 39.88, 31.76, 29.85, 29.18, 29.14, 23.79, 22.62, 14.11. HRMS (FD, $C_{37}H_{42}S_2Br_4$): calcd, 865.9456; found, 865.9450.

Synthesis of compound 3

To a solution of compound **1** (400 mg, 0.5 mmol) in dry THF (2 mL) under nitrogen was added isopropylmagnesium chloride lithium chloride complex solution (1.3 M in THF, 0.8 mL) dropwise at −78 °C. After stirring at −78 °C for 30 min, a solution of zinc chloride (157 mg, 1.15 mmol) in dry THF (1 mL) was added to the mixture at −78 °C. The reaction mixture was warmed to room temperature and stirred for 1 h to form the F-Zn intermediate. Then, a solution of 2,3-dibromo-6-undecylthieno[3,2-b]thiophene (565 mg, 1.5 mmol), $Pd(PPh_3)_2Cl_2$ (70 mg, 0.1 mmol) and SPhos (41 mg, 0.1 mmol) in dry toluene (6 mL) were added with the freshly prepared F-Zn solution slowly at room temperature. The mixture was refluxed for 16 h. After cooling to room temperature, the reaction mixture was quenched by NH_4Cl solution and extracted with dichloromethane (50 mL x 2) and water (50 mL). The collection organic layer was dried over anhydrous $MgSO_4$. After removing the solvent by reduced pressure, the residue was purified by silica gel chromatography with hexane as an eluent to get a light-yellow oil **3** (218 mg, 34%). ^{1}H NMR (400 MHz, $CDCl_3$): δ 8.03 (s, 2H), 7.44 (s, 2H), 7.07 (s, 2H), 2.74 (t, J = 7.6 Hz, 4H), 1.95 (m, 4H), 1.77 (m, 4H), 1.40-1.07 (m, 46H), 0.90-0.21 (m, 22H). ^{13}C NMR (100 MHz, $CDCl_3$): δ 150.45, 141.97, 140.07, 138.00, 137.97, 135.99, 133.48, 127.84, 124.96, 123.63, 122.02, 104.23, 55.62, 39.99, 32.06, 31.92,29.97, 29.79, 29.77, 29.72, 29.52, 29.50, 29.32, 29.29, 28.86, 22.84, 22.78, 14.28, 14.24. HRMS (FD, $C_{63}H_{86}S_4Br_4$): calcd, 1286.2351; found, 1286.2358.

Synthesis of FNT

To a mixture of compound **2** (330 mg, 0.38 mmol), sodium *tert*-butoxide (328 mg, 3.41 mmol), $Pd_2(dba)_3$ (69 mg, 0.08 mmol) and BINAP (189 mg, 0.3 mmol) in degassed *p*-xylene (4.5 mL) was added 2-butyl octan-1-amine (421 mg, 2.27 mmol). The mixture was stirred for 16 h at 135 °C. The reaction solution was filtered through celite and extracted with dichloromethane (25 mL x 2) and water (50 mL). The collection organic layer was dried over $MgSO_4$. After removing the solvent by reduced pressure, the residue was purified by column chromatography with dichloromethane/hexane (1/5, v/v) as the eluent to get a yellow oil **FNT** (108 mg, 31%). ^1H NMR (400 MHz, $CDCl_3$): δ 7.66 (s, 2H), 7.62 (s, 2H), 7.33 (d, J = 5.2 Hz, 2H), 7.05 (d, J = 5.2 Hz, 2H), 4.19 (d, J = 7.2 Hz, 4H), 2.19 (m, 2H), 2.05 (m, 4H), 1.26-0.84 (m, 70H), 0.75 (m, 4H). ^{13}C NMR (100 MHz, $CDCl_3$): δ 146.07, 143.02, 141.90, 137.32, 125.70, 121.36, 116.02, 112.71, 110.77, 100.01, 53.56, 49.79, 42.31, 38.47, 31.83, 31.48, 30.29, 29.64, 29.36, 28.75, 26.50, 23.92, 23.06, 22.64, 22.59, 14.14, 14.10, 14.05. HRMS (FD, $C_{61}H_{92}N_2S_2$): calcd, 916.6707; found, 916.6706.

Synthesis of FNTT

To a mixture of compound **3** (300 mg, 0.23 mmol), sodium *tert*-butoxide (201 mg, 2.09 mmol), $Pd_2(dba)_3$ (43 mg, 0.05 mmol) and BINAP (116 mg, 0.19 mmol) in degassed *p*-xylene (2.8 mL) was added 2-butyl octan-1-amine (258 mg, 1.39 mmol). The mixture was stirred for 18 h at 135 °C. The reaction solution was filtered through celite and extracted with dichloromethane (25 mL x 2) and water (50 mL). The collection organic layer was dried over anhydrous $MgSO_4$. After removing the solvent by reduced pressure, the residue was purified by column chromatography with dichloromethane/hexane (1/7, v/v) as an eluent to get a yellow oil **FNTT** (108mg, 33%). ^1H NMR (400 MHz, $CDCl_3$):

δ 7.67 (s, 2H), 7.58 (s, 2H), 6.97 (s, 2H), 4.28 (d, J = 6.8 Hz, 4H), 2.79 (t, J = 7.6 Hz, 4H), 2.27 (s, 2H), 2.08 (m, 4H), 1.83 (m, 4H), 1.45-1.05 (m, 80H), 0.90- 0.70 (m, 36H). ^{13}C NMR (100 MHz, $CDCl_3$): δ 143.59, 141.44, 141.05, 137.60, 137.25, 136.60, 122.95, 122.51, 119.49, 117.08, 112.37, 100.42, 68.11, 53.71, 39.22, 32.09, 31.97, 31.93, 29.86, 29.80, 29.77, 29.72, 29.64, 29.61, 29.52, 28.94, 28.88, 26.68, 23.21, 22.86, 22.76, 22.73, 14.28, 14.23, 14.18. HRMS (FD, $C_{87}H_{136}N_2S_4$): calcd, 1336.9592; found, 1336.9601.

Synthesis of FNT-CHO

$POCl_3$ (0.5 mL, 0.53 mmol) was added to DMF (1 mL) and stirred for 30 min at 0 ℃. To a solution of **FNT** (91.8 mg, 0.1 mmol) in 1,2-dichloroethane (12 mL) was added the above mixture dropwise and stirred for 8 h at 80 ℃. The reaction was poured into water and stirred for 30 min. The reaction solution was extracted with dichloromethane (25 mL x 2) and water (50 mL). The collection organic layer was dried over anhydrous $MgSO_4$. After removing the solvent by reduced pressure, the residue was purified by silica gel chromatography with ethyl acetate/hexane (1/5, v/v) as an eluent to get a yellow solid **FNT-CHO** (51 mg, 52%). ^{1}H NMR (400 MHz, $CDCl_3$): δ 9.97 (s, 2H), 7.73 (s, 2H), 7.69 (m, 4H), 4.23 (d, J = 6.8 Hz, 4H), 2.19 (m, 2H), 2.09 (m, 4H), 1.45-1.35 (m, 12H), 1.27-1.19 (m, 12H), 1.14-0.98 (m, 26H), 0.90-0.86 (m, 14H), 0.77-0.73 (m, 10H). ^{13}C NMR (100 MHz, $CDCl_3$): δ 183.18, 145.25, 144.20, 143.93, 142.95, 139.76, 125.01, 121.16, 118.30, 114.57, 100.75, 53.80, 49.91, 42.02, 38.49, 31.83, 31.76, 31.46, 30.07, 29.58, 29.29, 29.23, 28.73, 26.46, 23.91, 23.01, 22.60, 22.54, 14.09, 14.06, 14.00. HRMS (FD, $C_{63}H_{92}N_2O_2S_2$): calcd, 972.6606; found, 972.6602.

Synthesis of FNTT-CHO

$POCl_3$ (0.35 mL, 0.37 mmol) was added to DMF (0.75 mL) and stirred for 30 min at 0 ℃. To a solution of **FNTT** (100 mg, 0.08 mmol) in 1,2-dichloroethane (9 mL) was

added the above mixture dropwise and stirred for 16 h at 80 °C. The reaction was poured into water and stirred for 30 min. The reaction solution was extracted with dichloromethane (25 mL x 2) and water (50 mL). The collection organic layer was dried over anhydrous $MgSO_4$. After removing the solvent by reduced pressure, the residue was purified by silica gel chromatography with ethyl acetate/hexane (1/10, v/v) as an eluent to get a yellow oil **FNTT-CHO** (47 mg, 45%). ^1H NMR (400 MHz, $CDCl_3$): δ 10.02 (s, 2H), 7.64 (m, 4H), 3.14 (t, J = 7.6 Hz, 4H), 2.17 (m, 4H), 1.91 (m, 6H), 1.33-1.09 (m, 80H), 0.90-0.72 (m, 36H). ^{13}C NMR (100 MHz, $CDCl_3$): δ 181.70, 147.02, 143.92, 142.13, 142.10, 138.39, 137.03, 136.27, 128.91, 122.14, 121.99, 112.74, 101.36, 53.49, 38.92, 31.92, 31.82, 31.70, 31.57, 31.23, 30.27, 29.68, 29.62, 29.55, 29.43, 29.34, 23.02, 22.70, 22.61, 14.13, 14.07, 14.06, 13.99. HRMS (FD, $C_{89}H_{136}N_2O_2S_4$): calcd, 1392.9490; found, 1392.9497.

Synthesis of FNT-FIC

To a mixture of **FNT-CHO** (50 mg, 0.05 mmol) and 2-(5,6-difluoro-3-oxo-2,3-dihydro-1H-inden-1-ylidene)malononitrile (47 mg, 0.21 mmol) in $CHCl_3$/DMF (6.2 mL, 5/1, v/v) was added trimethylsilyl chloride (1.28 mL, 10.1 mmol). The reaction mixture was stirred for 16 h at 40 °C. The reaction solution was extracted with dichloromethane (50 mL x 2) and water (75 mL). The collection organic layer was dried over anhydrous $MgSO_4$. After removing the solvent by reduced pressure, the residue was purified by silica gel chromatography with dichloromethane/hexane (1/1, v/v) as an eluent to get a dark blue solid **FNT-FIC** (64 mg, 89%). ^1H NMR (400 MHz, $CDCl_3$): δ 8.98 (s, 2H), 8.57-8.53 (m, 2H), 7.91 (s, 2H), 7.79 (s, 2H), 7.73-7.68 (m, 4H), 4.23 (d, J = 6.4 Hz, 4H), 2.21 (m, 2H), 2.11 (m, 4H), 1.47-1.37 (m, 12H), 1.35-1.21 (m, 20H), 1.16-1.03 (m, 20H), 0.91-0.81 (m, 14H), 0.77-0.74 (m, 10H). ^{13}C NMR (150 MHz, $CDCl_3$): δ 185.59, 158.86, 155.29, 153.66, 147.32, 146.16, 145.41,

141.94,138.91, 138.63, 136.68, 135.56, 134.85, 124.21, 121.85, 121.42, 115,82, 115.01, 114.87, 114.56, 112.65, 112.53, 101.39, 69.07, 53.93, 50.27, 41.80, 38.51, 32.06, 31.77, 31.66, 30.09, 29.61, 29.29, 29.23, 28.82, 26.57, 24.15, 23.03, 22.61, 22.57, 14.00, 13.95. HRMS (FD, $C_{87}H_{96}N_6O_2F_4S_2$): calcd, 1396.6978; found, 1396.6983.

Synthesis of FNTT-FIC

To a mixture of **FNTT-CHO** (40 mg, 0.03 mmol) and 2-(5,6-difluoro-3-oxo-2,3-dihydro-1H-inden-1-ylidene)malononitrile (27 mg, 0.11 mmol) in $CHCl_3$/DMF (3.5 mL, 5/1, v/v) was added trimethylsilyl chloride (0.7 mL, 5.5 mmol). The reaction mixture was stirred for 16 h at 40 ℃. The reaction solution was extracted with dichloromethane (50 mL x 2) and water (75 mL). The collection organic layer was dried over anhydrous $MgSO_4$. After removing the solvent by reduced pressure, the residue was purified by silica gel chromatography with dichloromethane/hexane (1/1, v/v) as an eluent to get a dark blue solid **FNTT-FIC** (37 mg, 71%). ^{1}H NMR (400 MHz, $CDCl_3$): δ 8.88 (s, 2H), 8.60 (m, 2H), 7.70-7.65 (m, 6H), 2.93 (m, 4H), 2.23 (m, 4H), 1.98 (m, 2H), 1.73(m, 8H), 1.33-1.18 (m, 68H), 1.17-1.11 (m, 16H), 0.91-0.81 (m, 12H), 0.77-0.74 (m, 16H). ^{13}C NMR (100 MHz, $CDCl_3$): δ 185.86, 158.72, 155.52, 153.85, 153.05, 152.91, 144.82, 144.04, 143.66, 139.68, 136.67, 135.59, 134.60, 134.42, 133.55, 126.59, 122.22, 119.11, 115.26, 114.63, 113.59, 112.17, 111.99, 101.69, 67.80, 53.61, 39.62, 32.03, 31.92, 31.83, 31.64, 30.75, 29.72, 29.65, 29.61, 29.49, 29.33, 22.70, 22.64, 22.62, 14.16, 14.12, 14.02. HRMS (FD, $C_{113}H_{140}N_6O_2F_4S_4$): calcd, 1816.9862; found, 1816.9816.

2. UV-vis spectrophotometer

UV-vis absorption spectra were measured on HP8453 UV-vis spectrophotometer. The neat films were prepared by spin-coating chloroform solution of the materials (10 mg

mL^{-1}) at 3000 rpm for 30 s on the quartz substrate.

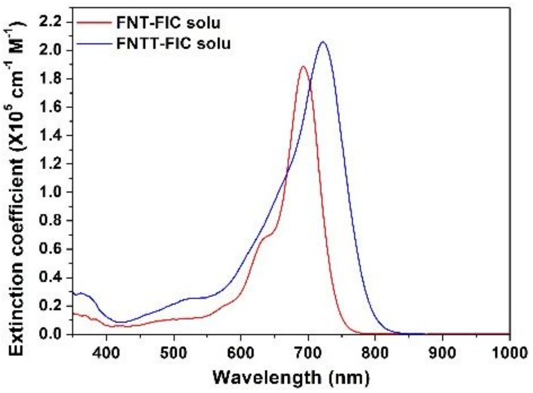

Figure S1. Absorption spectra of FNT-FIC and FNTT-FIC in chloroform solution with the extinction coefficient plotted on the Y-axis.

3. Cyclic voltammetry (CV) characteristics

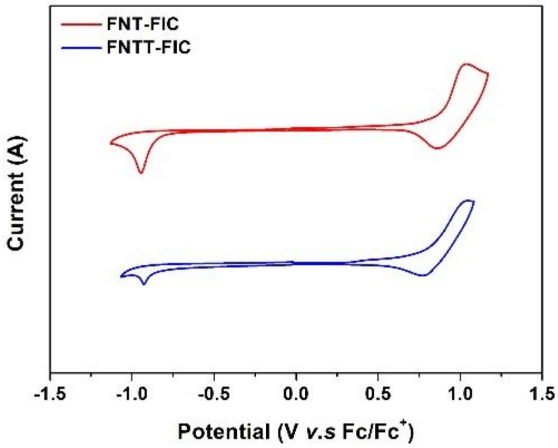

Figure S2. Cyclic voltammogram of FNT-FIC and FNTT-FIC in thin films at a scan rate of 100 mV s^{-1}.

CV was conducted on a CH instruments electrochemical analyzer. A carbon glass was used as the working electrode and an Ag/AgCl electrode as the reference electrode, while 0.1 M tetrabutylammonium hexafluorophosphate in acetonitrile was the electrolyte. CV curves were calibrated using ferrocene as the standard, whose HOMO energy level is set at −4.8 eV with respect to zero vacuum level. The HOMO energy

levels were obtained from the equation $E_{\mathrm{HOMO}} = -|E_{\mathrm{ox}}^{\mathrm{onset}} - E\mathrm{onset\ ferrocene} + 4.8|$ eV (S1). The LUMO energy levels were obtained from the equation $E_{\mathrm{LUMO}} = -|E_{\mathrm{red}}^{\mathrm{onset}} - E\mathrm{onset\ ferrocene} + 4.8|$ eV (S2).

4. Density functional theory (DFT) calculation of frontier molecular orbitals

Computational model compounds for FNT-FIC and FNTT-FIC, in which all alkyl chains are simplified into methyl groups, were calculated using Gaussian09 suite 15 at the B3LYP/6-311G(d,p) level.

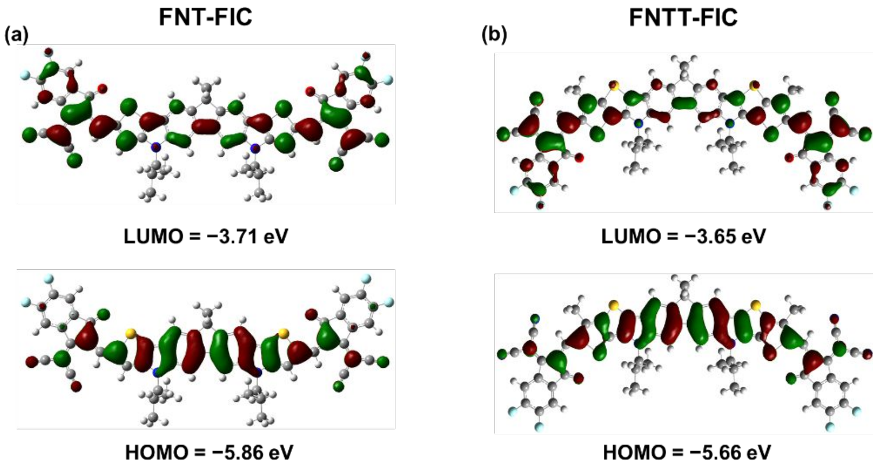

Figure S3. Theoretical electron distribution of (a) FNT-FIC and (b) FNTT-FIC. calculated by DFT at B3LYP/6-311G(d,p) level.

5. Fabrication and Characterization of OPV Devices

The fabrication of the inverted devices follows the procedures: The ITO-coated glass substrates were cleaned by ultrasonic cleaner in detergent, DI-water, acetone and isopropyl alcohol for 10 min, respectively, and subsequently treated with UV-ozone for 45 min. The ZnO layer was prepared by the ZnO precursor (diethyl Zinc) solution in THF and spin-coated onto the pre-treated ITO-coated glass. The chlorobenzene solution of PM6:FNT-FIC and *o*-xylene solution of PM6:FNTT-FIC in an different weight ratio were prepared and stired 12 h at 60 °C. Active layers were formed by spin-

coating on top of the ZnO/ITO substrate. Moreover, the substrates were thermally annealed at different temperature and time in the glove box. Finally, the MoO_3 layer (7 nm) and silver anode (150 nm) were deposited by thermal evaporation at a pressure below 10^{-6} torr. The devices without encapsulation were characterized in ambient condition. Current-voltage characteristics were measured by a Keithley 2400 SMU under the irradiation of AM 1.5G San-Yi solar simulator with JIS AAA spectrum. The characteristics of the solar cells were optimized by testing approximately 10 cells. IPCE spectra were measured using a lock-in amplifier with a current preamplifier under short-circuit conditions with illumination by monochromatic light from a 250 W quartz-halogen lamp (Osram) passing through a monochromator (Spectral Products CM110).

Table S1. Summary of binary device parameters based on nonacyclic ladder structure of NFAs reported in the literature

Year	Acceptor	Donor	V_{oc} (V)	J_{sc} (mA/cm^2)	FF (%)	PCE (%)	Ref
2018	FNIC1	PTB7-Th	0.774	19.97	66.4	10.3	3
2018	FNIC2	PTB7-Th	0.741	23.93	73.4	13.0	3
2019	DTFT9-FIC	PBDB-T	0.86	19.01	58.61	9.58	4
2019	BTTFIC	PBDB-T	0.935	17.74	54.3	9.00	5
2019	CZTT-IC	PBDB-T	0.97	17.26	58.97	9.87	6
2019	CZTT-4F	PM6	0.94	19.73	65.06	12.07	6
2019	IN-4F	PM6	0.870	21.8	69.2	13.0	7
2019	INPIC-4F	PBDB-T	0.852	21.9	69.8	12.7	8

2019	INPIC-EH	PBDB-T	0.837	20.7	68.4	11.7	8
2019	INPIC-BO	PBDB-T	0.839	20.0	66.8	10.9	8
2020	FCTT-FIC	PM6	0.90	19.49	69.73	12.23	9
2020	DTTC-4F	PM6	0.95	21.66	67.60	13.89	10
2020	DTTC-4Cl	PM6	0.92	22.64	74.04	15.42	10
2021	DTTC-4F-C8	PM6	0.98	19.19	70.79	13.36	11
2021	DTTC-4Cl-C9	PM6	0.95	20.50	67.23	13.17	11
2021	GL1	PBDB-T	0.761	22.59	66.5	11.43	12
2021	F-2Cl	PBDB-T	0.743	18.92	73.4	10.31	12
2021	BP6T-4F	PM6	0.91	11.00	64.20	6.43	13
2021	ABP5T-4F	PM6	0.88	24.64	73.01	15.81	13
2024	FNTT-FIC	PM6	0.96	19.64	64.52	12.15	This work

6. Space-charge limited current (SCLC) characteristics

The hole-only and electron-only devices were fabricated by employing the following device structure: ITO/PEDOT:PSS/active layer/Au for holes and ITO/ZnO/active layer/Al for electrons. The mobilities were obtained by taking current-voltage curves and fitting the results to the equation listed below:

$$J = \frac{9\varepsilon_0\varepsilon_r\mu V^2}{8L^3} \qquad \text{(S3)}$$

where J is the current density, ε_0 the vacuum permittivity, ε_r the relative dielectric constant, μ the mobility, V the voltage, and L the film thickness.

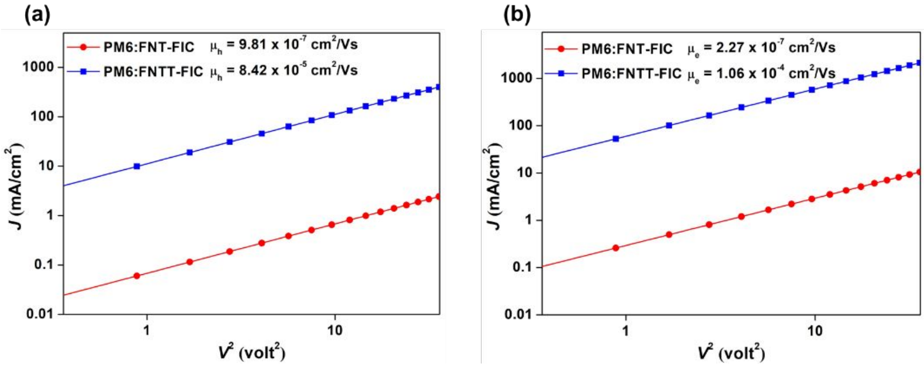

Figure S4. *J-V* curves of the (a) hole-only and (b) electron-only devices of PM6:FNT-FIC and PM6:FNTT-FIC thin films.

7. GIWAXS film measurements

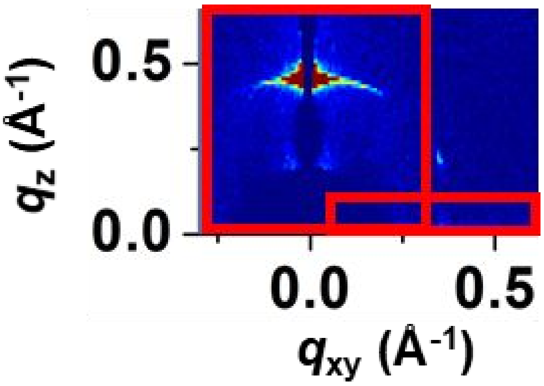

Figure S5. 2D GIWAXS in the scattering vector space presentation. The GIWAXS profiles were extracted along the areas highlighted by the two rectangle long the in-plane q_{xy} and out-of-plane q_z directions as indicated.

Synchrotron grazing incidence wide-angle X-ray scattering (GIWAXS) of the 25A coherent X-ray scattering beamline of the Taiwan Photon Source (TPS) was used to investigate the molecular packing of the neat NFA and blend films with PM6. The corresponding 1D scattering profiles extracted from the selected zones along the in-plane (q_{xy}) and out-of-plane (q_z) directions of the 2D GIWAXS patterns collected with an Eiger X 1M detector for the sample thin films are illustrated in Figure S5**.** The microbeam (ca. a couple tens of μm) incident angle was set to 0.03°; the sample-to-detector distance was 70.0 mm. 2D GIWAXS patterns were further converted to the

scattering vector space, with q_{xy} and q_z representing the scattering vector components respectively along the in-plane and out-of-plane directions;[14-15] after the conversion, there is a missing wedge of no diffraction information available in the vertical direction of each 2D GIWAXS pattern.[14-15]

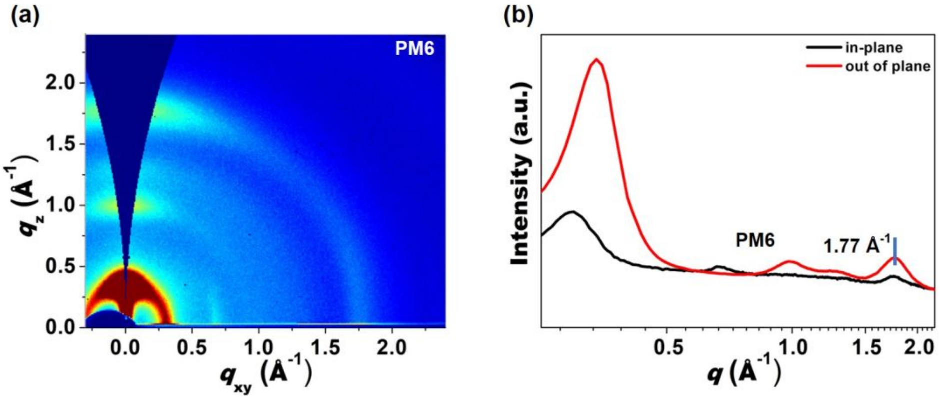

Figure S6. 2D GIWAXS patterns of (a) PM6 and (b) corresponding scattering plots of neat film along the in-plane and out-of-plane directions.

8. Contact angle measurements

PM6, FNT-FIC and FNTT-FIC films were formed by spin-coating on the clean silicon dioxide substrates. Contact angle were obtained from contact angle (CA) goniometer (FTA 125).

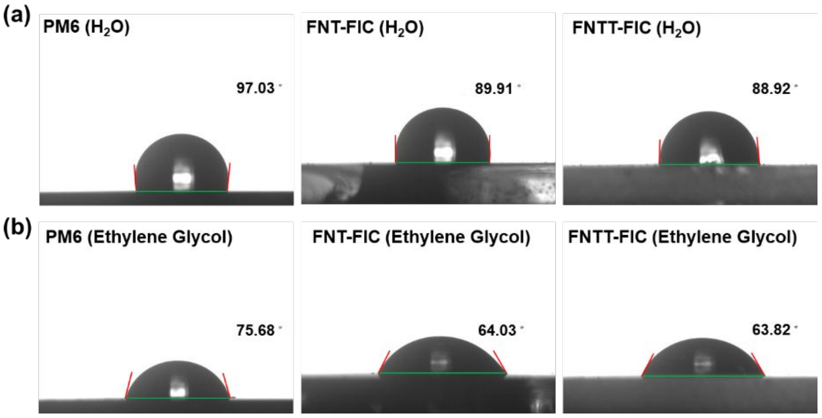

Figure S7. Contact angle images of PM6 and NFA thin films with (a) water and (b) ethylene glycol droplet on top.

9. References

1. Lee, C.-H.; Lai, Y.-Y.; Cheng, S.-W.; Cheng, Y.-J. Synthesis and Supramolecular Assembly of Pentacyclic Dithienofluorene and Diselenophenofluorene Derivatives. *Org. Lett.* **2014**, *16*, 936-939.

2. Xue, Y.-J.; Lai, Z.-Y.; Lu, H.-C.; Hong, J.-C.; Tsai, C.-L.; Huang, C.-L.; Huang, K.-H.; Lu, C.-F.; Lai, Y.-Y.; Hsu, C.-S.; Lin, J.-M.; Chang, J.-W.; Chien, S.-Y.; Lee, G.-H.; Jeng, U.-S.; Cheng, Y.-J. Unraveling the Structure-property-performance Relationships of Fused-ring Nonfullerene Acceptors: Toward a C-Shaped ortho-Benzodipyrrole-based Acceptor for Highly Efficient Organic Photovoltaics. *J. Am. Chem. Soc.* **2024,** *146*, 833-848.

3. Wang, J.; Zhang, J.; Xiao, Y.; Xiao, T.; Zhu, R.; Yan, C.; Fu, Y.; Lu, G.; Lu, X.; Marder, S. R.; Zhan, X. Effect of Isomerization on High-performance Nonfullerene Electron Acceptors. *J. Am. Chem. Soc.* **2018,** *140*, 9140-9147.

4. Cao, F.-Y.; Huang, P.-K.; Su, Y.-C.; Huang, W.-C.; Chang, S.-L.; Hung, K.-E.; Cheng, Y.-J. Forced Coplanarity of Dithienofluorene-based Non-fullerene Acceptors to Achieve High-Efficiency Organic Solar Cells. *J. Mater. Chem. A* **2019,** *7*, 17947-17953.

5. Fan, X.; Gao, J.; Wang, W.; Xiao, S.; Zhan, C.; Lu, X.; Zhang, Q. Ladder-type Nonacyclic Arene Bis(thieno[3,2-b]thieno)cyclopentafluorene as a Promising Building Block for Non-fullerene Acceptors. *Chem. Asian J.* **2019,** *14*, 1814-1822.

6. Wang, H.; Zhang, Z.; Yu, J.; Liu, X.; Qu, S.; Guang, S.; Tang, W. Nonacyclic Carbazole-based Non-fullerene Acceptors Enable over 12% Efficiency with Enhanced Stability for Organic Solar Cells. *J. Mater. Chem. A* **2019,** *7*, 21903-21910.

7. Su, D.; Pan, M.-A.; Liu, Z.; Lau, T.-K.; Li, X.; Shen, F.; Huo, S.; Lu, X.; Xu, A.; Yan, H.; Zhan, C. A Trialkylsilylthienyl Chain-substituted Small-molecule Acceptor with Higher LUMO Level and Reduced Band Gap for Over 16% Efficiency Fullerene-free Ternary Solar Cells. *Chem. Mater.* **2019,** *31*, 8908-8917.

8. Feng, H.; Song, X.; Zhang, M.; Yu, J.; Zhang, Z.; Geng, R.; Yang, L.; Liu, F.; Baran, D.; Tang, W. Side Chain Engineering on Dithieno[3,2-b:2,3-d]pyrrol Fused Electron Acceptors for Efficient Organic Solar Ccells. *Mater. Chem. Front.* **2019,** *3*, 702-708.

9. Xue, Y.-J.; Cao, F.-Y.; Huang, P.-K.; Su, Y.-C.; Cheng, Y.-J. Isomeric Effect of Fluorene-based Fused-ring Electron Acceptors to Achieve High-efficiency Organic Solar Cells. *J. Mater. Chem. A* **2020,** *8*, 5315-5322.

10. Chen, T.-W.; Peng, K.-L.; Lin, Y.-W.; Su, Y.-J.; Ma, K.-J.; Hong, L.; Chang, C.-C.; Hou, J.; Hsu, C.-S. A Chlorinated Nonacyclic Carbazole-based Acceptor Affords over 15% Efficiency in Organic Solar Cells. *J. Mater. Chem. A* **2020,** *8*, 1131-1137.

11. Karapala, V. K.; Chen, T.-W.; Ma, K.-J.; Lu, P.-L.; Su, Y.-J.; Fu, W.-D.; Shih, H.-M.; Lu, S.-J.; Lee, T.-Y.; Chang, C.-F.; Chen, J.-T.; Hsu, C.-S. Exploring Ternary Organic Solar Cells for the Improved Efficiency of 16.5% with the Compatible Nonacyclic Carbazole-based Nonfullerene Acceptors as the Third Component. *ACS Appl. Energy Mater.* **2021,** *4*, 2847-2855.

12. Li, S.; Sun, Y.; Zhou, B.; Fu, Q.; Meng, L.; Yang, Y.; Wang, J.; Yao, Z.; Wan, X.; Chen, Y. Concurrently Improved J_{sc}, Fill Factor, and Stability in a Ternary Organic Solar Cell Enabled by a C-shaped Non-fullerene Acceptor and Its Structurally Similar Third Component. *ACS Appl. Mater. Interfaces* **2021,** *13*, 40766-40777.

13. Gao, W.; Fan, B.; Qi, F.; Lin, F.; Sun, R.; Xia, X.; Gao, J.; Zhong, C.; Lu, X.; Min, J.; Zhang, F.; Zhu, Z.; Luo, J.; Jen, A. K.-Y. Asymmetric Isomer Effects in Benzo[c][1,2,5]thiadiazole-fused Nonacyclic Acceptors: Dielectric Constant and Molecular Crystallinity Control for Significant Photovoltaic Performance Enhancement. *Adv. Funct. Mater.* **2021,** *31*, 2104369.

14. Jiang, Z. GIXSGUI: A MATLAB Toolbox for Grazing-incidence X-ray Scattering Data Visualization and Reduction, and Indexing of Buried Three-dimensional Periodic Nanostructured Films. *J. Appl. Cryst.* **2015**, 48, 917.

15. Baker, J. L.; Ji,son, L. H.; Mannsfeld, S.; Volkman, S.; Yin, S.; Subramanian, V.; Salleo, A.; Alivisatos, A. P.; Toney, M. F. Quantification of Thin Film Crystallographic Orientation Using X-ray Diffraction with an Area Detector *Langmuir* **2010**, *26*, 9146.

10. ^{1}H and ^{13}C NMR spectra

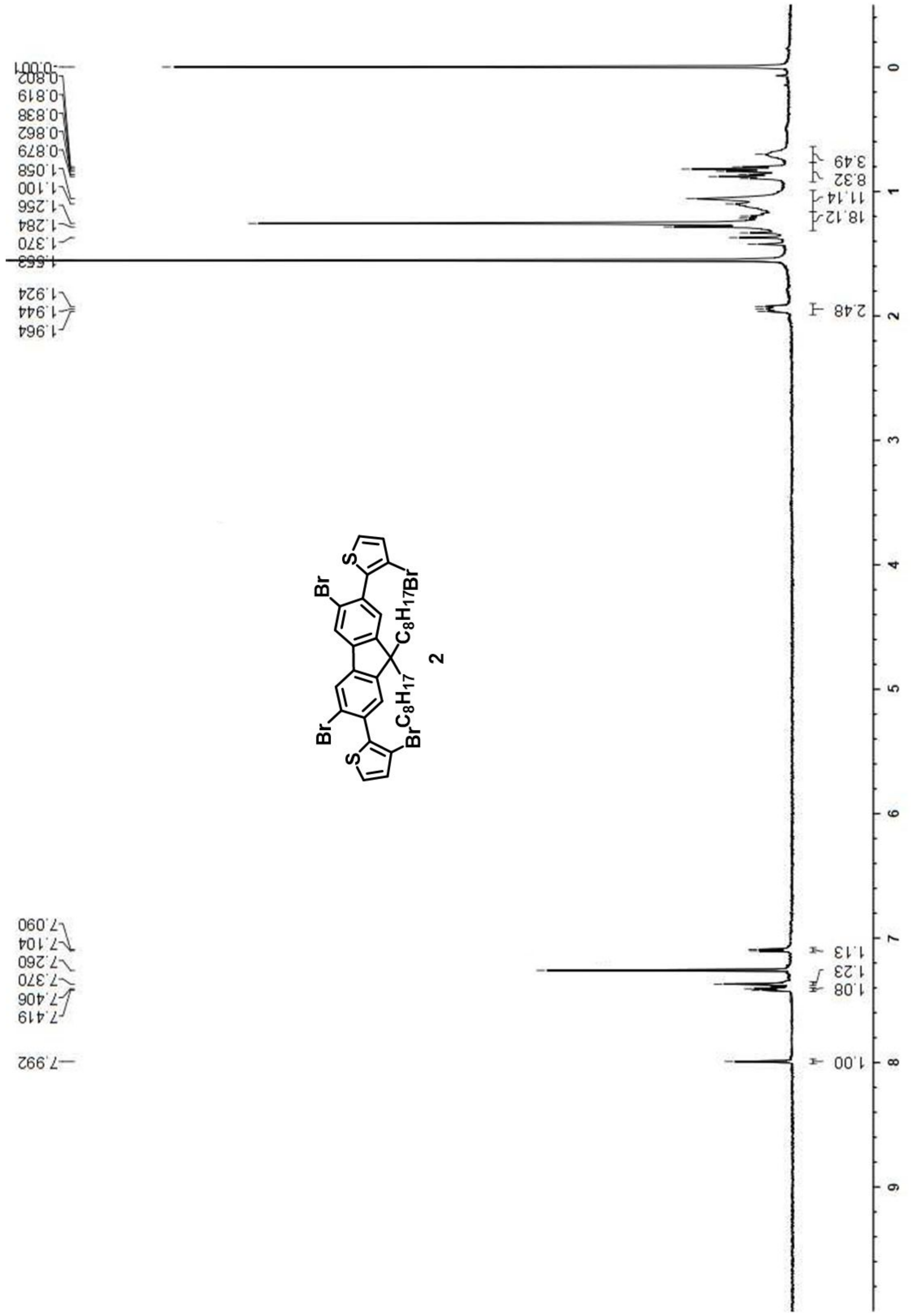

Figure S8. ^{1}H spectrum of compound 2.

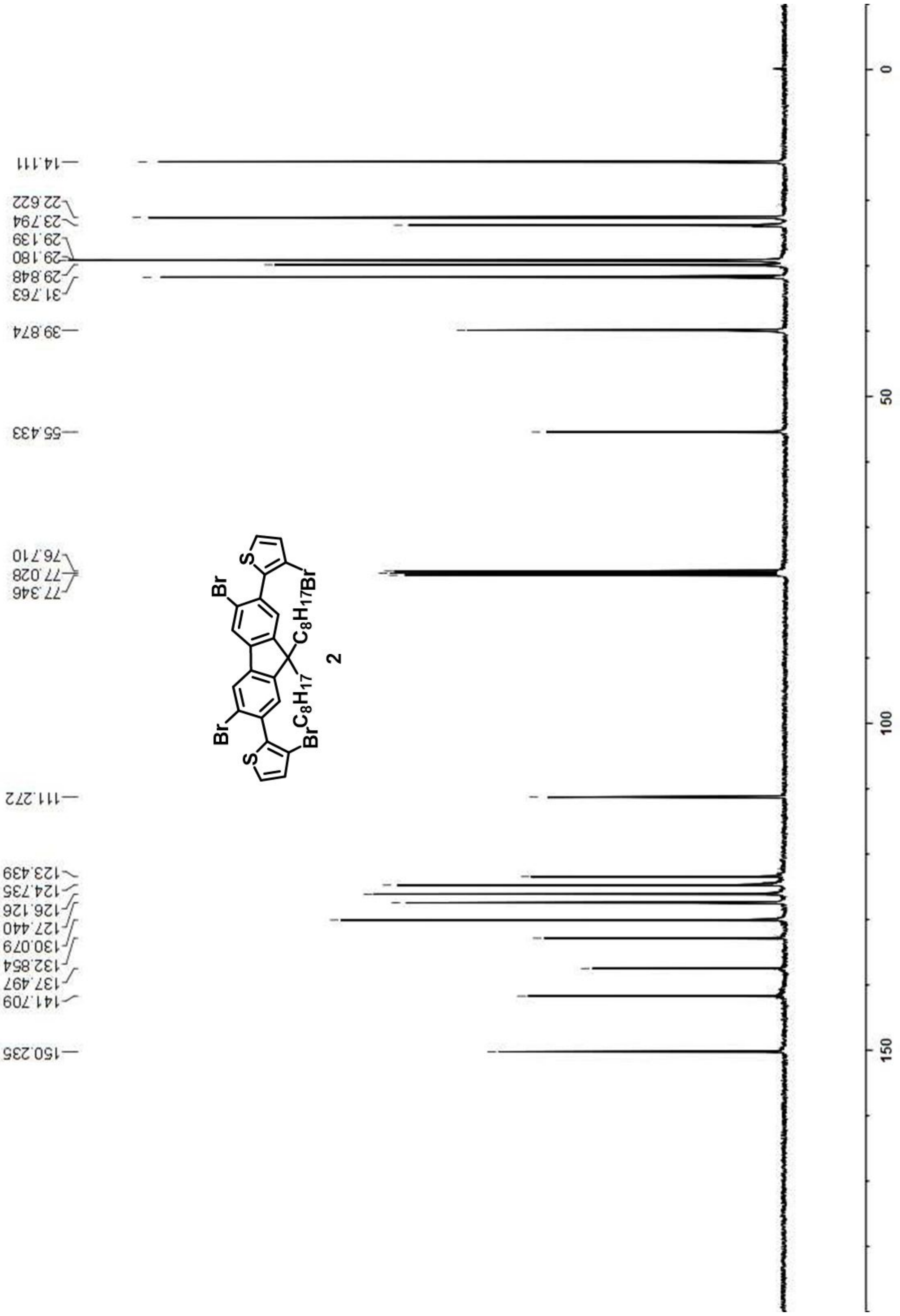

Figure S9. ^{13}C spectrum of compound 2.

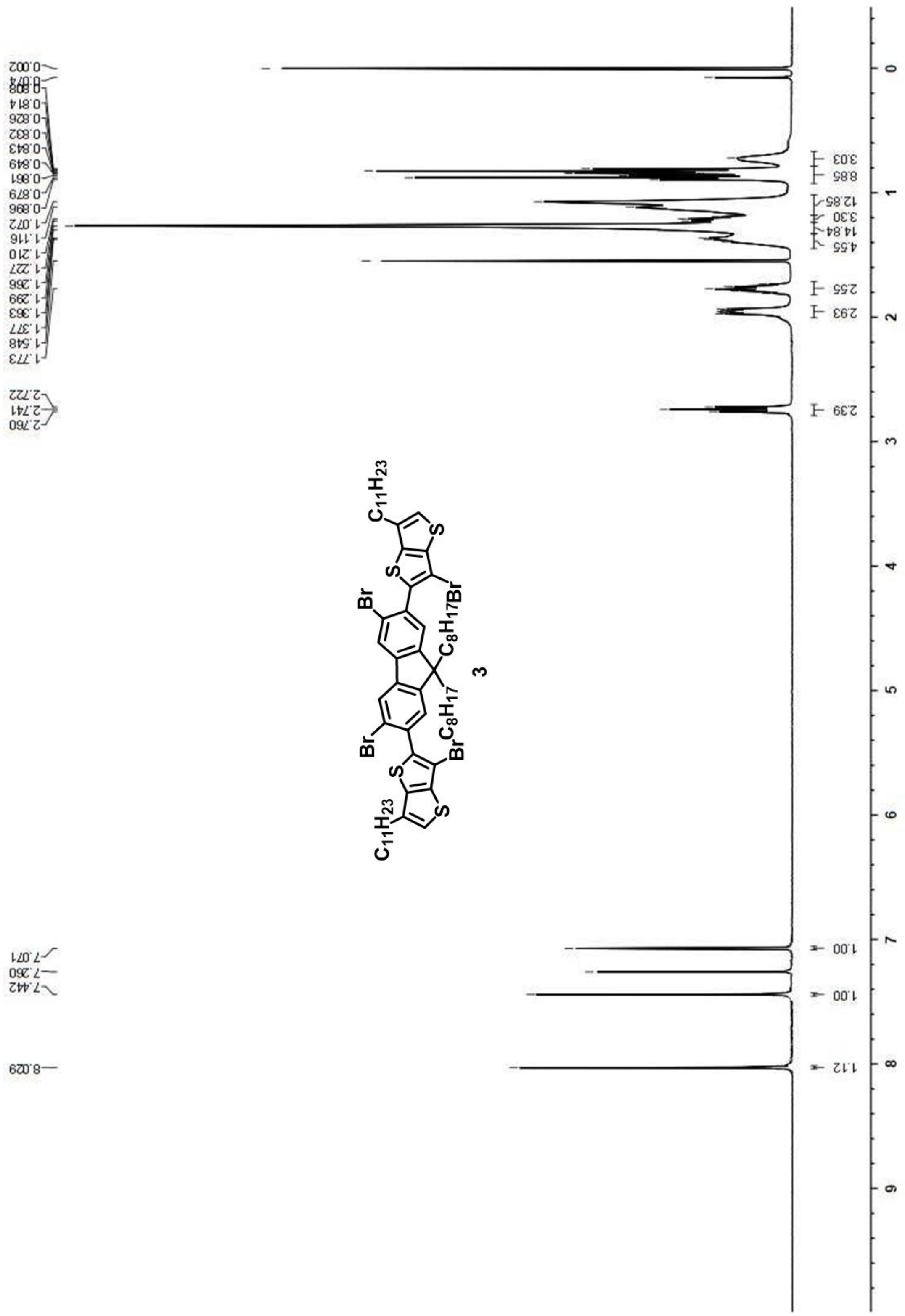

Figure S10. ^{1}H spectrum of compound 3.

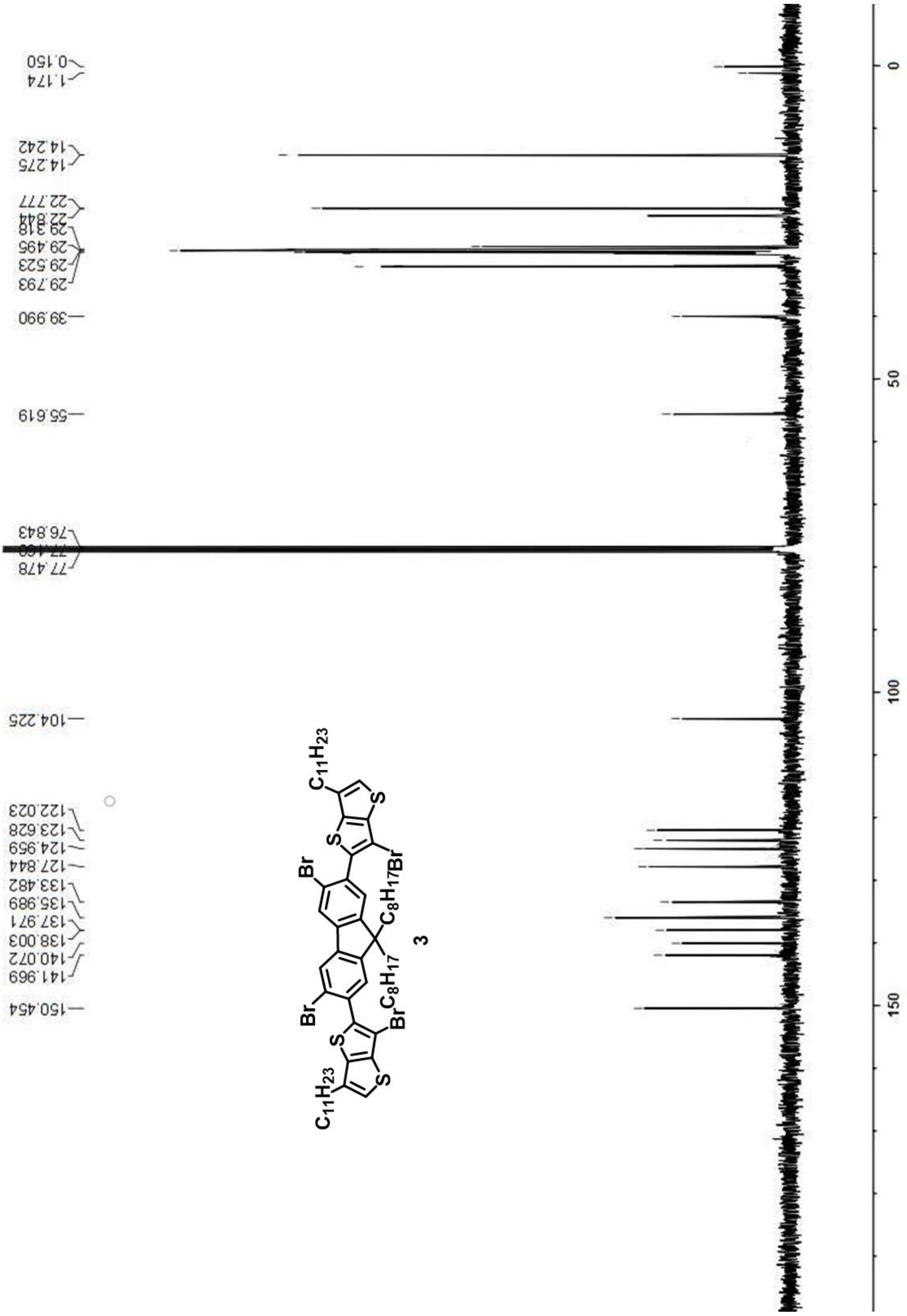

Figure S11. ^{13}C spectrum of compound 3.

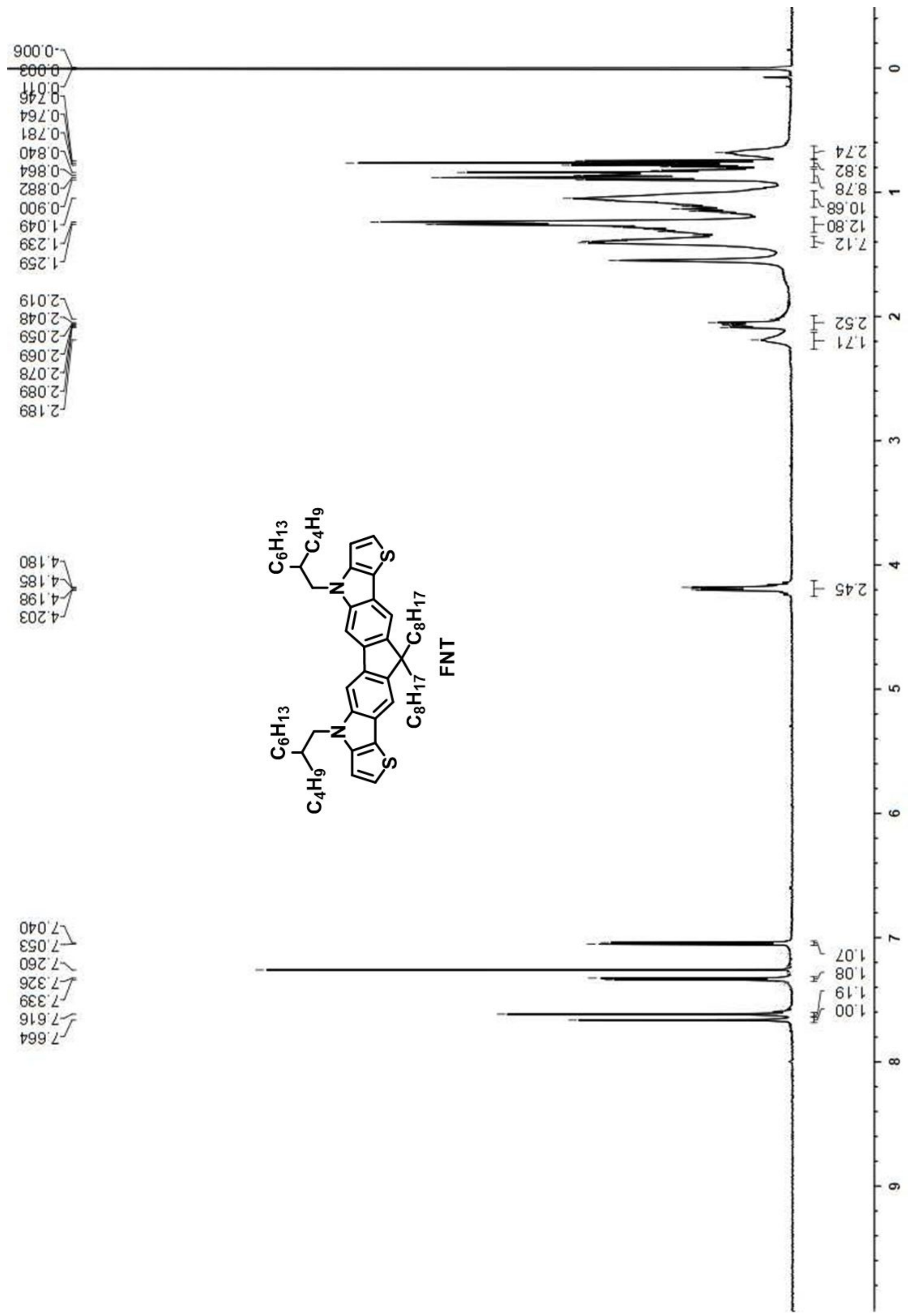

Figure S12. ^{1}H spectrum of FNT.

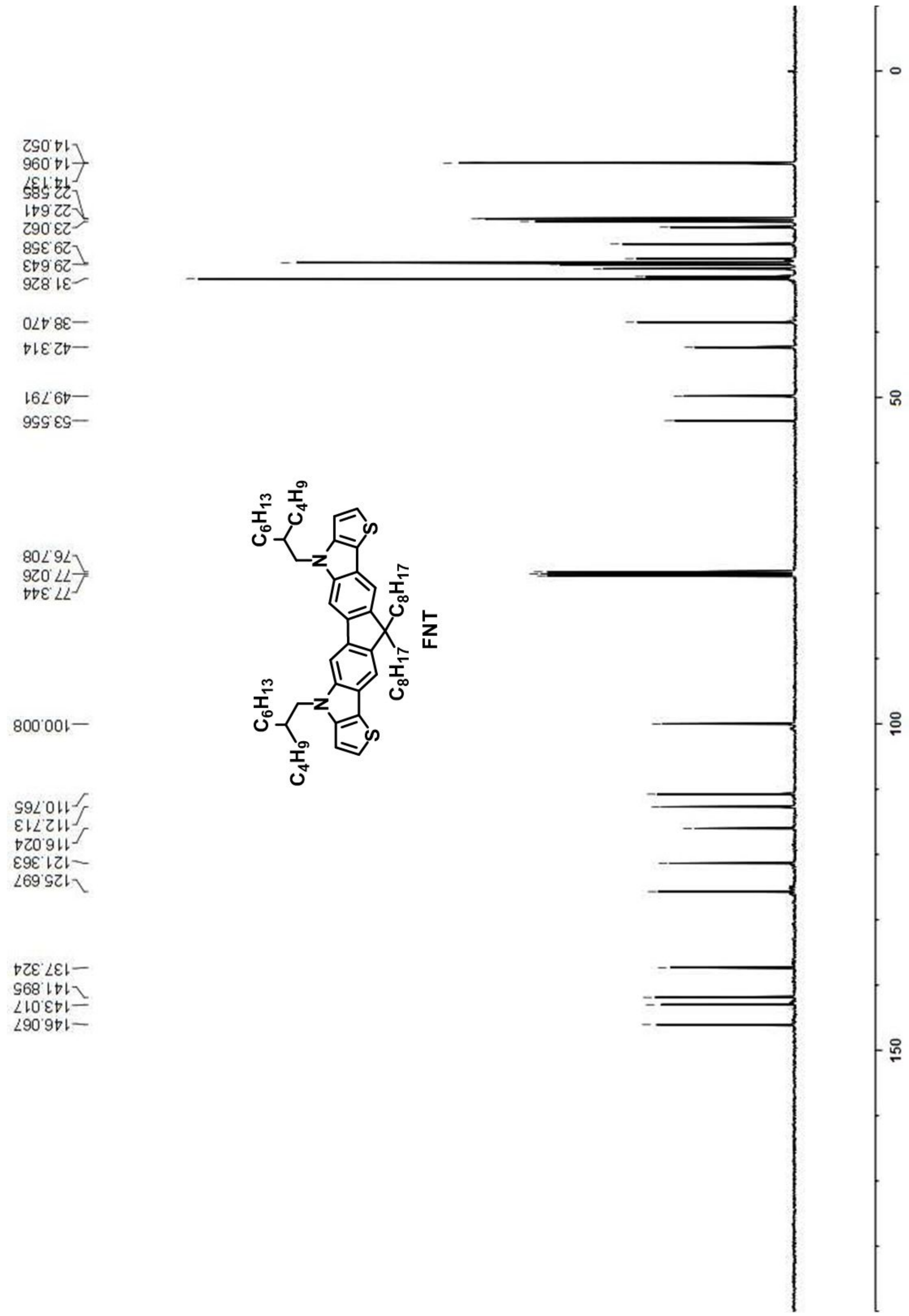

Figure S13. ^{13}C spectrum of FNT.

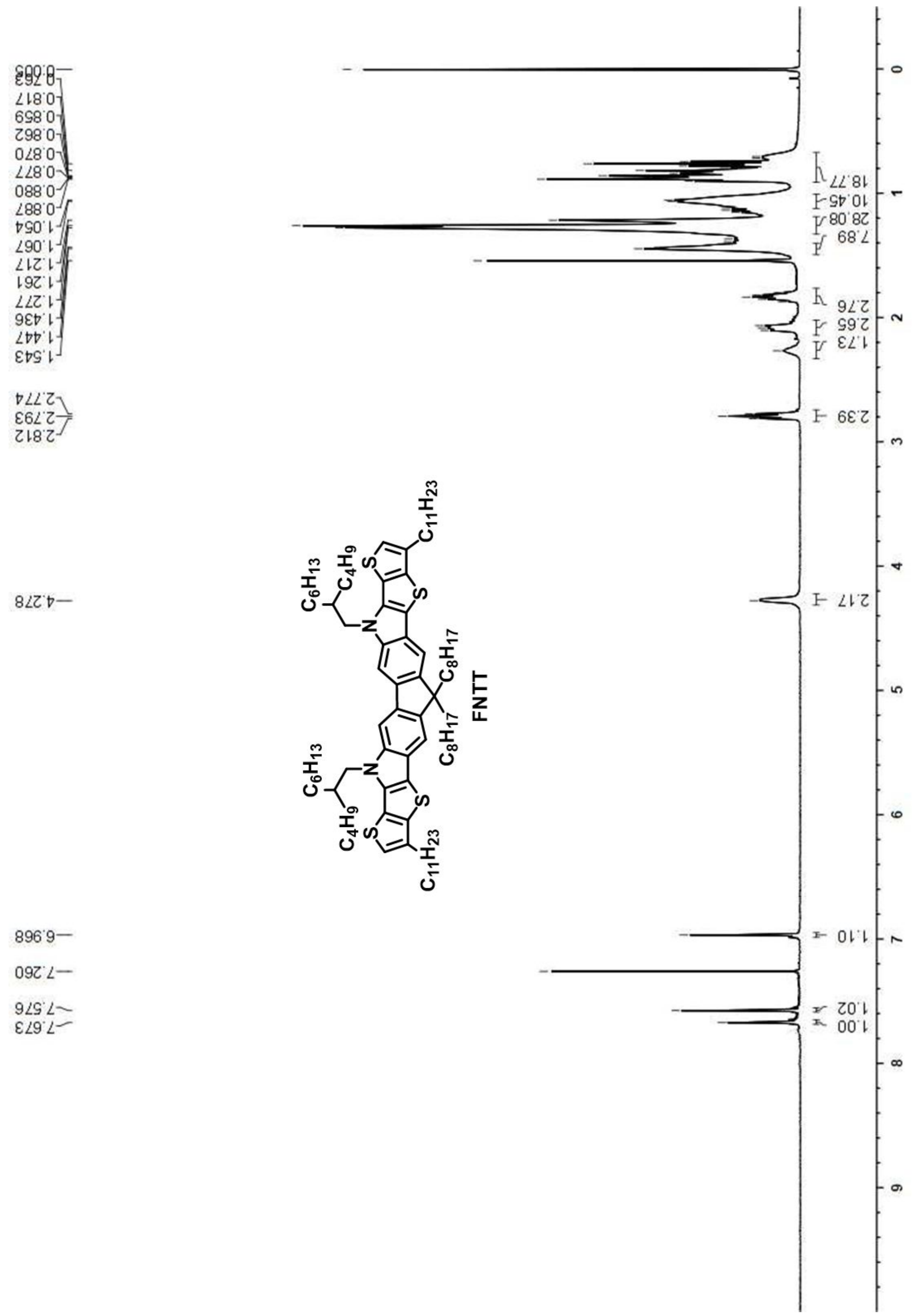

Figure S14. ^{1}H spectrum of FNTT.

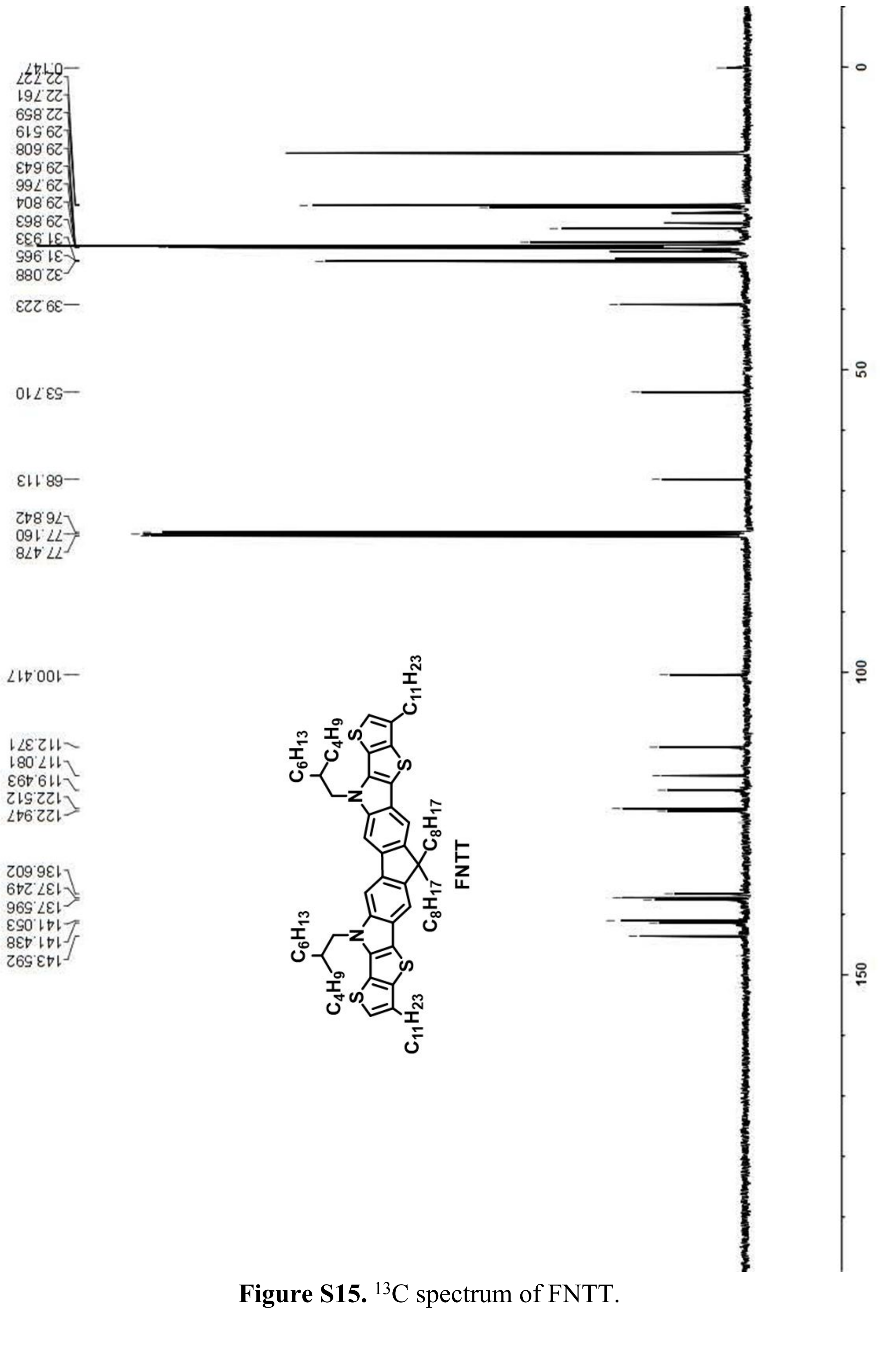

Figure S15. ^{13}C spectrum of FNTT.

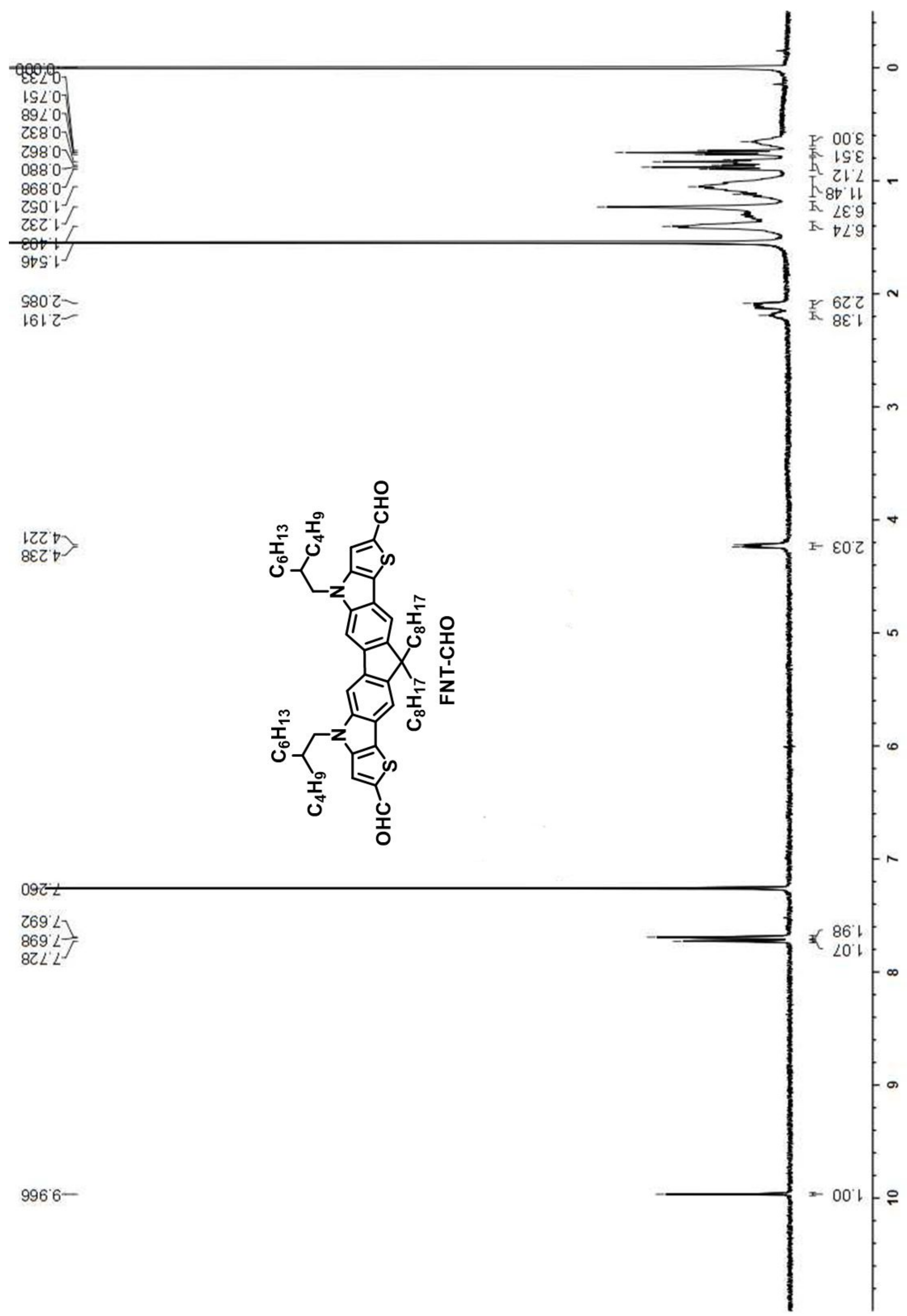

Figure S16. ^{1}H spectrum of FNT-CHO.

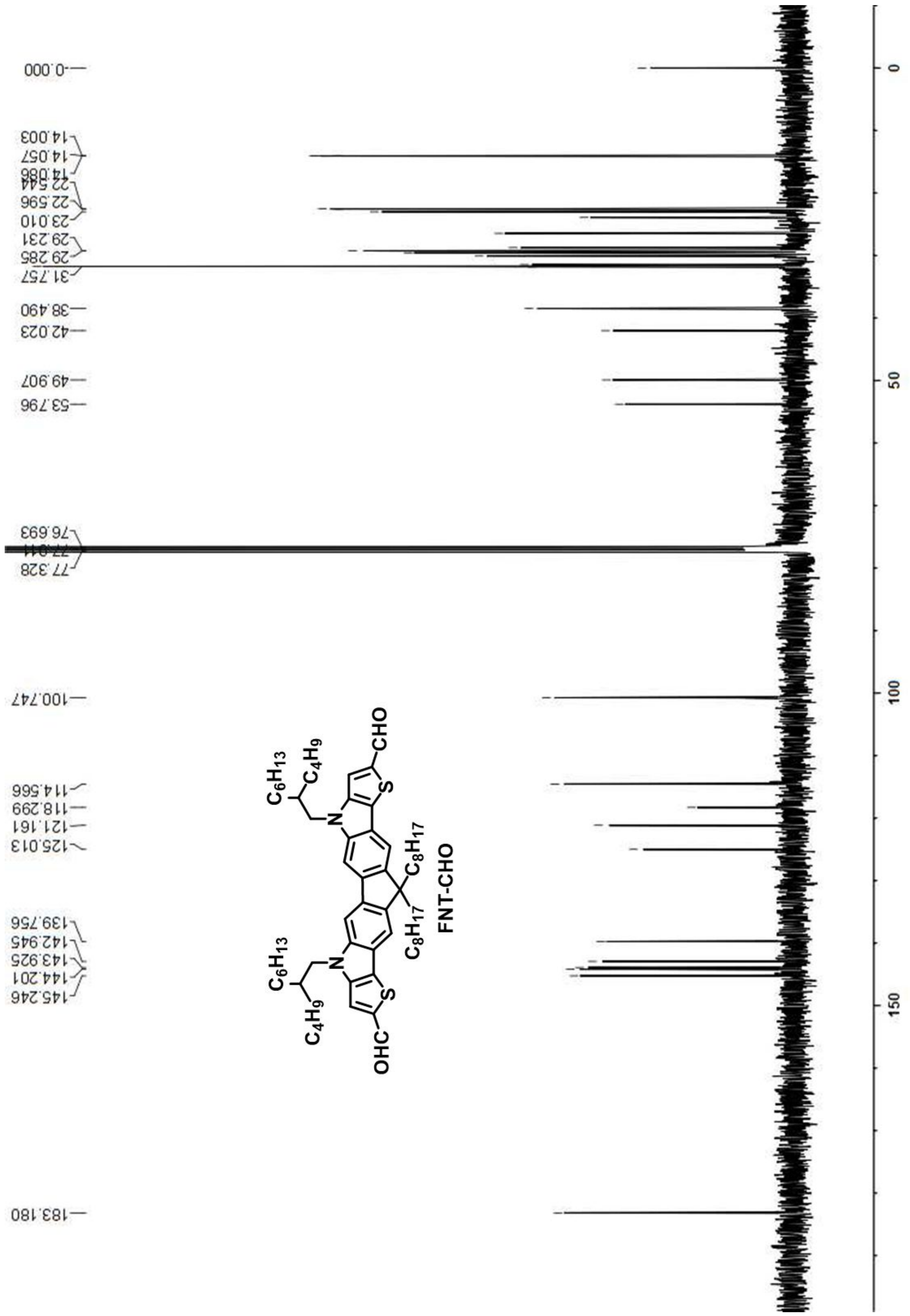

Figure S17. ^{13}C spectrum of FNT-CHO.

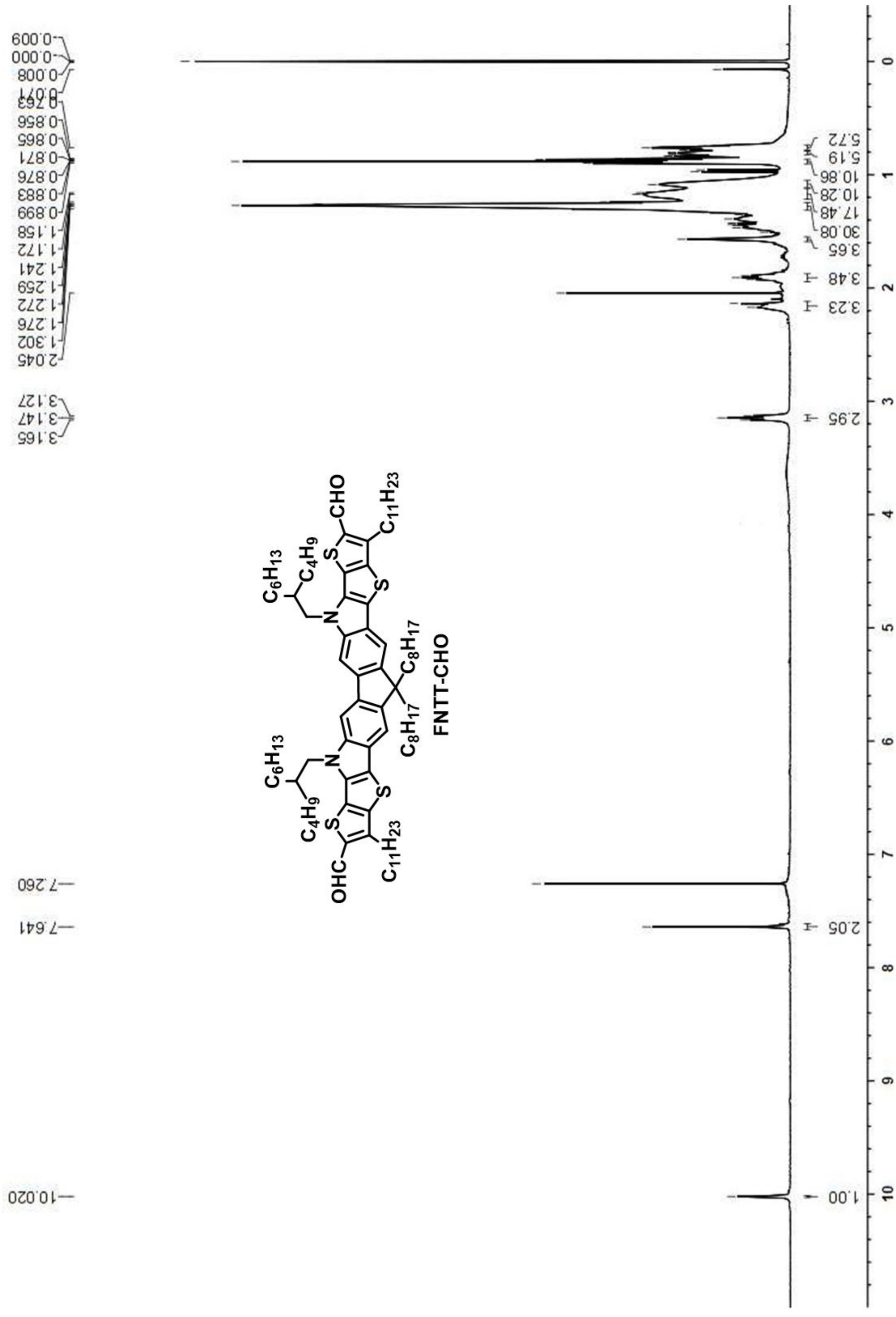

Figure S18. ^{1}H spectrum of FNTT-CHO.

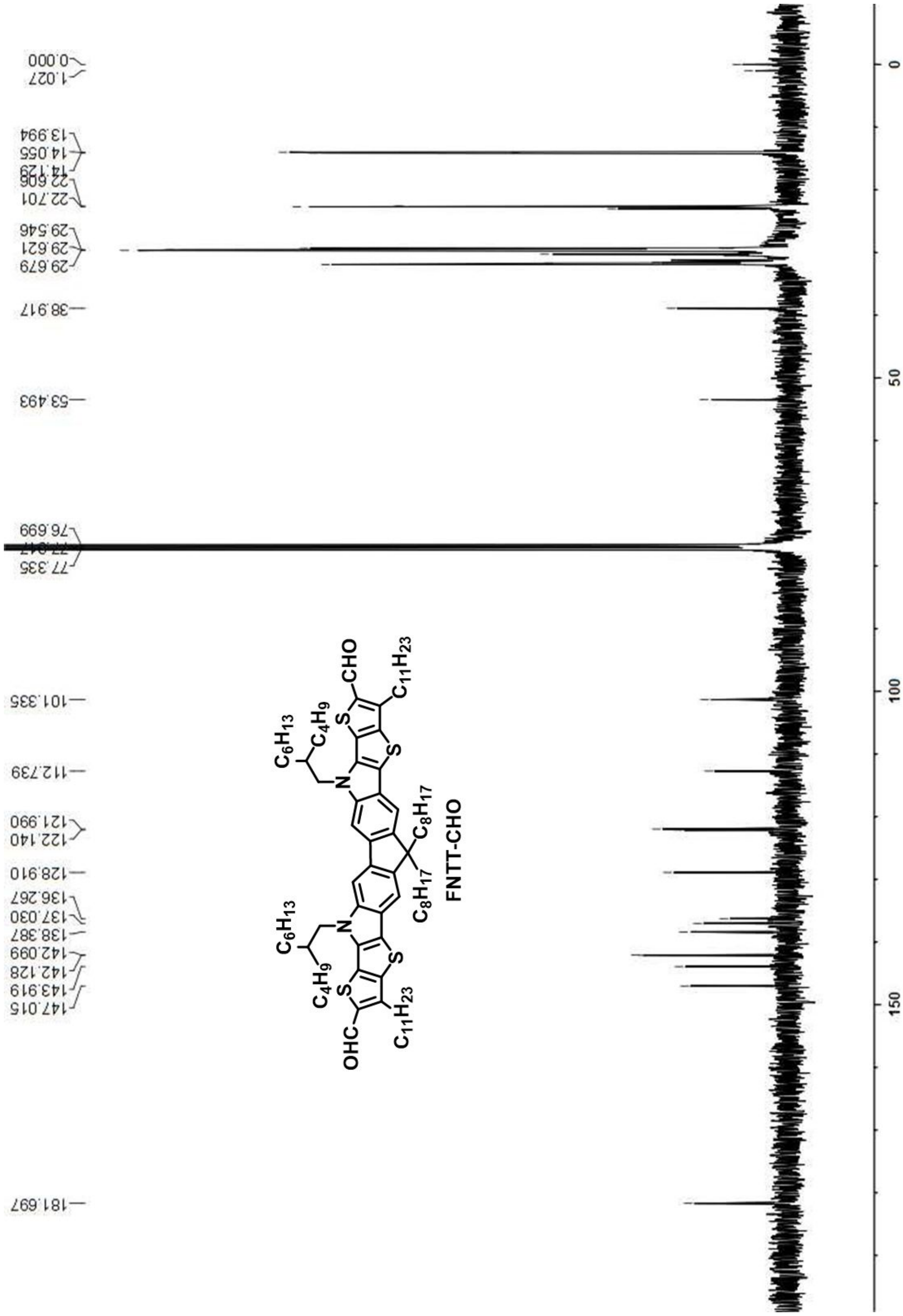

Figure S19. ^{13}C spectrum of FNTT-CHO.

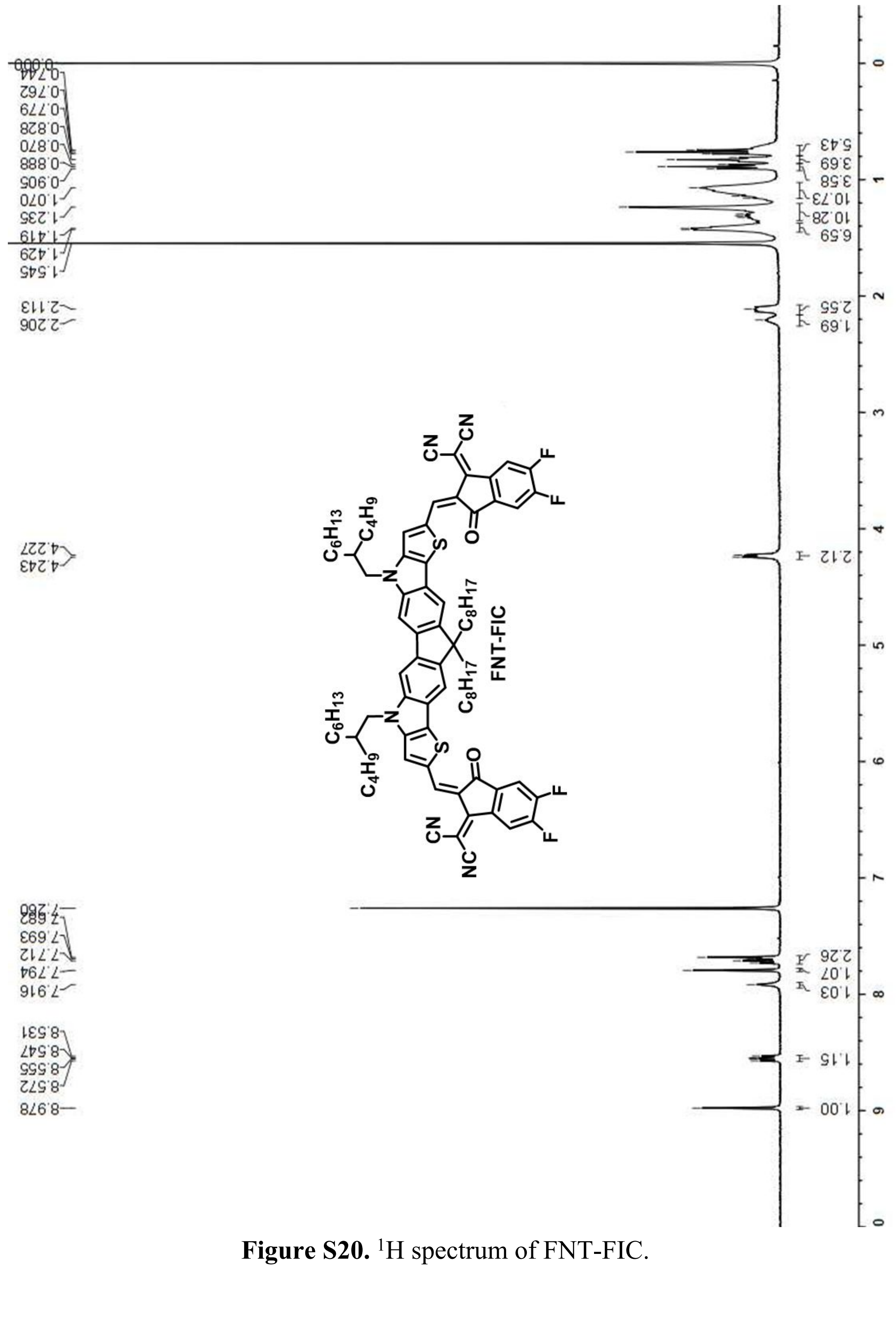

Figure S20. ^{1}H spectrum of FNT-FIC.

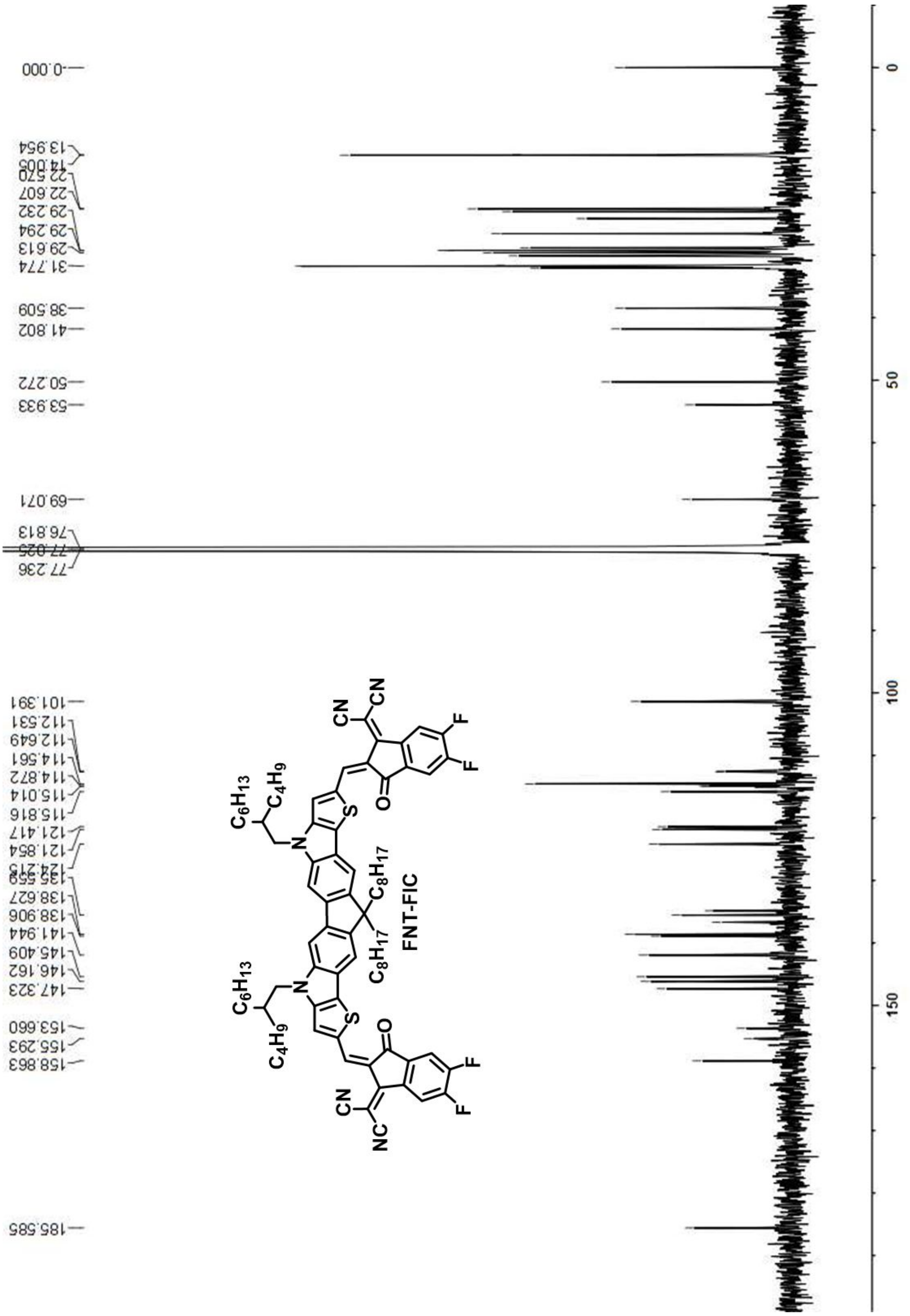

Figure S21. ^{13}C spectrum of FNT-FIC.

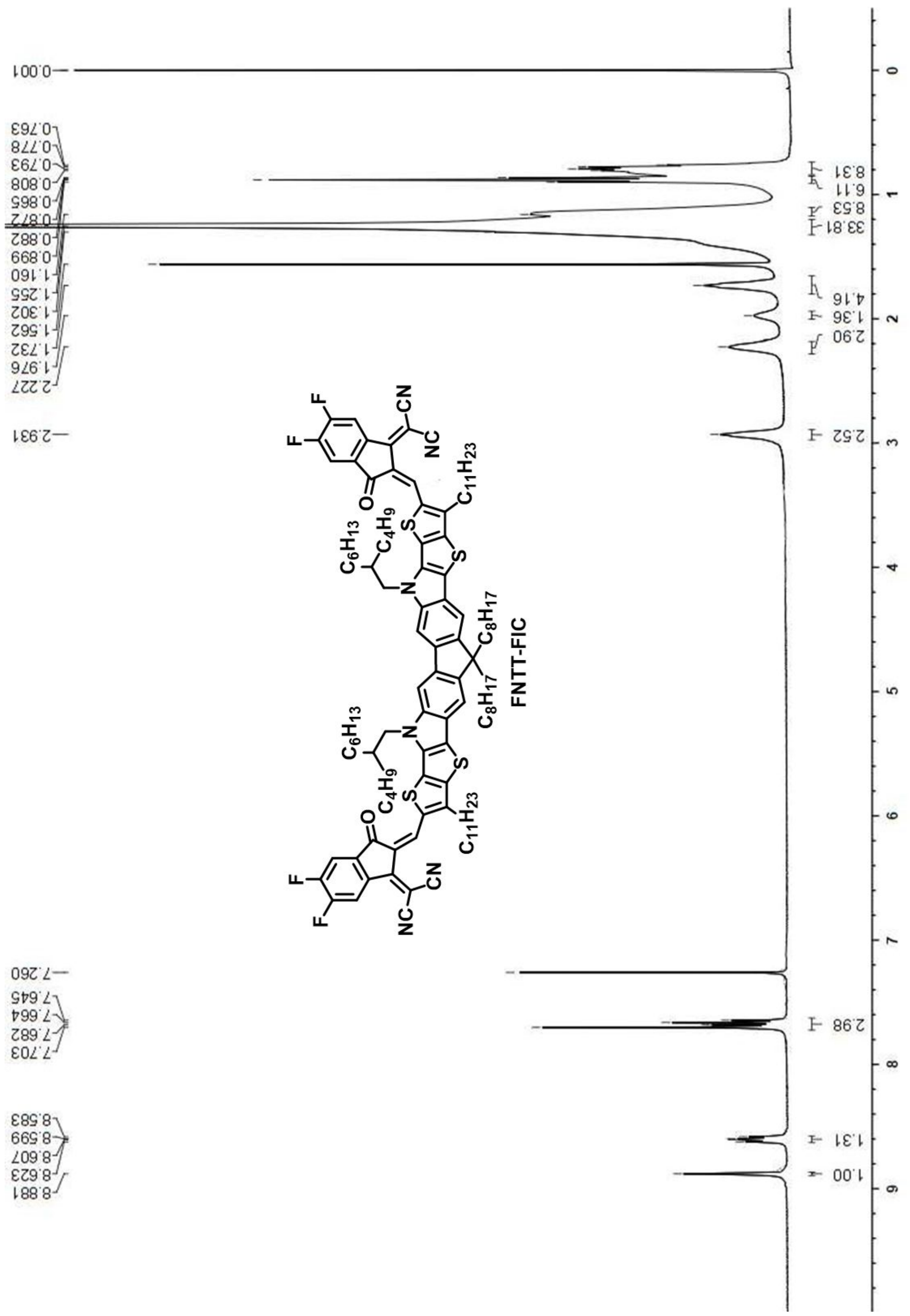

Figure S22. ^{1}H spectrum of FNTT-FIC.

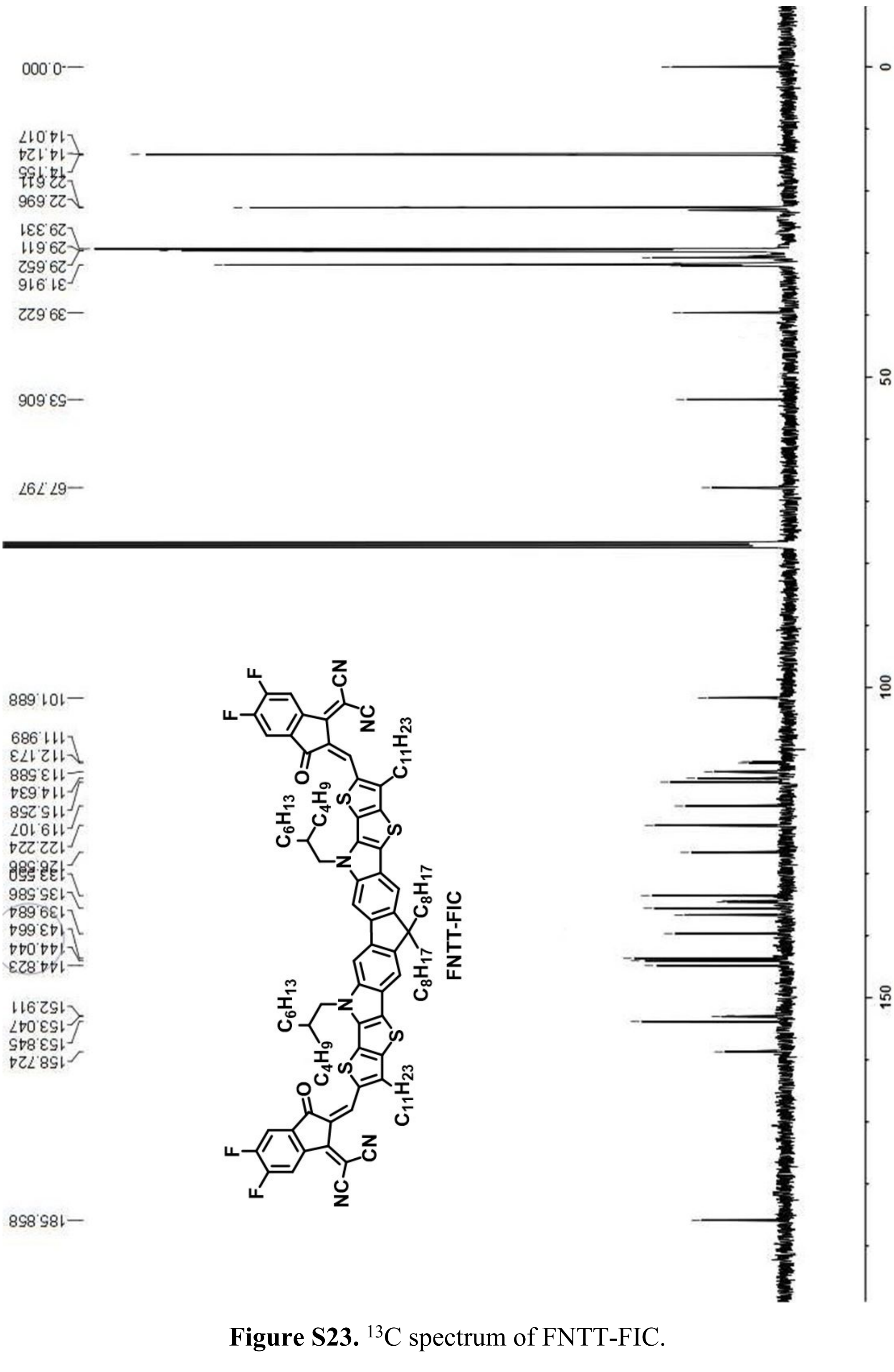

Figure S23. ^{13}C spectrum of FNTT-FIC.
